# Camel milk preservation: matrix effects across conventional and emerging technologies

**DOI:** 10.3389/fnut.2026.1990078

**Published:** 2026-10-23

**Authors:** Dadaie Daihi, Fouad Achemchem, Fadwa El Kindoussy, Oumayma Boukria, Poliana Mendes de Souza

**Affiliations:** 1 College of Agriculture and Environmental Sciences (CAES), African Sustainable Agriculture Research Institute (ASARI), University Mohammed VI Polytechnic (UM6P), Laayoune, Morocco; 2 Higher School of Technology, Laboratory of Engineering Sciences and Energy Management (LASIME), Ibn Zohr University, Agadir, Morocco

**Keywords:** camel milk, industrial implementation, matrix effects, non-thermal processing, preservation, process validation, shelf life

## Abstract

Camel milk supports food security, nutrition and rural economies across arid regions, while its commercial development is expanding towards refrigerated products, fermented foods, powders and functional ingredients. This transition exposes a preservation problem that differs from that of conventional dairy chains. Variable conditions before processing determine the initial microbial and enzymatic load, whereas the distinctive organisation of camel-milk proteins, minerals and dispersed phases alters treatment delivery, physical stability and quality retention. This critical narrative review integrates matrix knowledge with direct evidence on established and emerging preservation processes. Scopus, Web of Science Core Collection, PubMed, CAB Abstracts and Google Scholar were searched for relevant studies available up to August 2026. The evidence reveals no simple hierarchy of preservation technologies. Cooling, controlled fermentation, pasteurisation and drying remain the practical foundation of camel-milk processing, but their performance is bounded by raw-milk history and by matrix responses that include aggregation, weak gel formation, oxidation and loss of powder stability. These constraints have stimulated interest in lower-temperature and non-thermal approaches that may widen the interval between microbial control and unacceptable product modification. High-pressure processing currently offers the most developed pathogen-specific evidence within this group, while the reported performance of UV-C and ultrasound remains closely tied to reactor geometry, energy distribution and temperature history. Other emerging approaches are supported mainly by physicochemical observations or evidence transferred from non-camel matrices. Across all process classes, nominal equipment settings and immediate reductions in background microbiota provide an incomplete account of preservation performance. Reliable interpretation requires the treatment delivered within the product to be considered together with matrix composition, microbial target, quality boundaries and behaviour after processing. Camel-milk preservation should therefore be approached as a matrix-dependent process-design problem rather than as the selection of an individual technology.

## 1 Introduction

Camel milk plays an important role in food security, nutrition, and rural livelihoods across arid and semi-arid regions of Africa and Asia, where camel production remains viable under climatic and feed constraints that limit conventional dairy farming. Global production has increased steadily, reaching an estimated 4.1 million tonnes in 2023, with Eastern Africa accounting for most recorded output, although informal production and trade remain inadequately represented in official statistics (1). In Kenya, Somalia, Ethiopia, the Sahel, the Arabian Peninsula, and parts of Central Asia, camel milk is traditionally consumed fresh or fermented, while increasingly organised value chains are supporting the development of pasteurised milk, yogurt, cheese, milk powders, and ingredients for wider distribution (1–3). As these value chains expand, longer intervals between milking, processing, distribution, and consumption place greater demands on microbial safety, product stability, and shelf-life management, making preservation a critical determinant of commercial viability.

Beyond its importance in resource-constrained production systems, camel milk has attracted growing interest because of its distinctive nutritional composition and bioactive constituents. Its protein, lipid, mineral, and vitamin profiles, together with lactoferrin, immunoglobulins, lactoperoxidase, and bioactive peptides, have stimulated the development of functional foods and dairy alternatives (4–8). Camel milk also lacks β-lactoglobulin, a major allergenic whey protein in bovine milk, although this compositional distinction does not establish universal hypoallergenicity or justify assumptions regarding clinical tolerance (9, 10). Preserving the nutritional, bioactive, and technological attributes of camel milk while ensuring microbiological safety presents a particular challenge because its protein organisation and colloidal structure influence responses to thermal, pressure-based, optical, and acoustic treatments.

This challenge is compounded by the conditions under which camel milk is produced and handled. Production remains largely distributed across pastoral and peri-urban systems, where milking hygiene, water quality, container sanitation, ambient temperature, and delays before refrigeration vary considerably. Investigations in North Africa have documented variations in raw-milk quality associated with handling and hygiene practices, while studies in Kenya and Somaliland have highlighted persistent limitations in collection, transportation, and food-safety management (3, 11–14). Raw camel milk may contain coliforms, staphylococci, spore-forming bacteria, and zoonotic or foodborne pathogens. Although endogenous antimicrobial systems can inhibit or delay microbial growth under certain conditions, their presence does not ensure the microbiological safety of untreated milk (15–20). Consequently, inadequate control during collection and distribution can accelerate spoilage, compromise sensory and technological quality, increase product rejection, and shorten the period during which milk remains suitable for processing or consumption. These conditions also influence the initial microbial and enzymatic loads presented to subsequent preservation treatments, thereby affecting their performance.

Beyond variability in raw-milk quality, the physicochemical characteristics of camel milk impose additional constraints on processing. Similarities in gross protein and fat contents between camel and bovine milk do not translate into equivalent technological behaviour. Camel milk is characterised by a relatively high proportion of β-casein, low κ-casein abundance, larger casein micelles, and a whey-protein fraction dominated by α-lactalbumin and lacking β-lactoglobulin (21–25). These compositional and structural differences contribute to distinct responses during heating, coagulation, gel formation, and concentration (26–31). Consequently, processing conditions established for bovine milk cannot be transferred directly to camel milk without considering potential effects on protein aggregation, sedimentation, gel structure, flavour, and product stability. Although emerging non-thermal technologies offer opportunities to reduce heat-associated deterioration, their performance is also influenced by matrix properties, including optical opacity, viscosity, composition, and interactions between the product and processing equipment. Preservation efficacy therefore depends not only on the selected technology but also on the interaction between milk characteristics, treatment delivery, microbial susceptibility, and the quality requirements of the intended product.

Several reviews have addressed important aspects of camel-milk processing and preservation. Berhe et al. (32) examined conventional processing challenges and opportunities for product development, while Ho et al. (22) reviewed nutritional properties, heat stability, and technological applications. Hamed et al. (33) focused on antimicrobial constituents, fermented products, and shelf life, whereas Mazumder et al. (34) discussed emerging non-thermal processing technologies. More recently, Elsahoryi et al. (35) examined physicochemical deterioration, preservation of bioactive compounds, and non-thermal approaches. Collectively, these contributions provide a substantial foundation for understanding camel-milk composition, processing behaviour, and preservation strategies. However, a critical issue remains insufficiently integrated across conventional and emerging technologies: the extent to which reported experimental outcomes support reliable and transferable preservation claims. Differences in milk composition, initial microbial conditions, treatment delivery, experimental design, and analytical endpoints complicate comparisons among studies. Moreover, reductions in indigenous microbial populations do not necessarily demonstrate control of defined foodborne hazards, while favourable quality measurements immediately after processing may not predict product stability during storage or performance under industrial conditions. These distinctions are essential for interpreting the available evidence and identifying preservation strategies that can be reliably implemented beyond laboratory settings.

Against this background, the present review critically examines conventional and emerging camel-milk preservation technologies through an integrated assessment of milk-matrix characteristics, processing conditions, microbial efficacy, and product-quality responses. Particular emphasis is placed on distinguishing nominal processing parameters from the treatment delivered to the product, experimental reductions in microbial populations from validated safety or shelf-life outcomes, and laboratory-scale observations from evidence supporting industrial application. By evaluating the available literature within this framework, the review identifies the principal factors governing preservation performance, assesses the strength and transferability of existing evidence, and establishes research priorities for the development and validation of reliable, matrix-adapted preservation processes for camel milk and its products.

## 2 Review approach

A critical narrative review was conducted to examine camel-milk preservation, focusing on the relationships among milk-matrix characteristics, processing conditions, microbial control, and product quality. The literature search, study selection, evidence classification, and synthesis approach are summarised in Figure 1.

**Figure 1:**
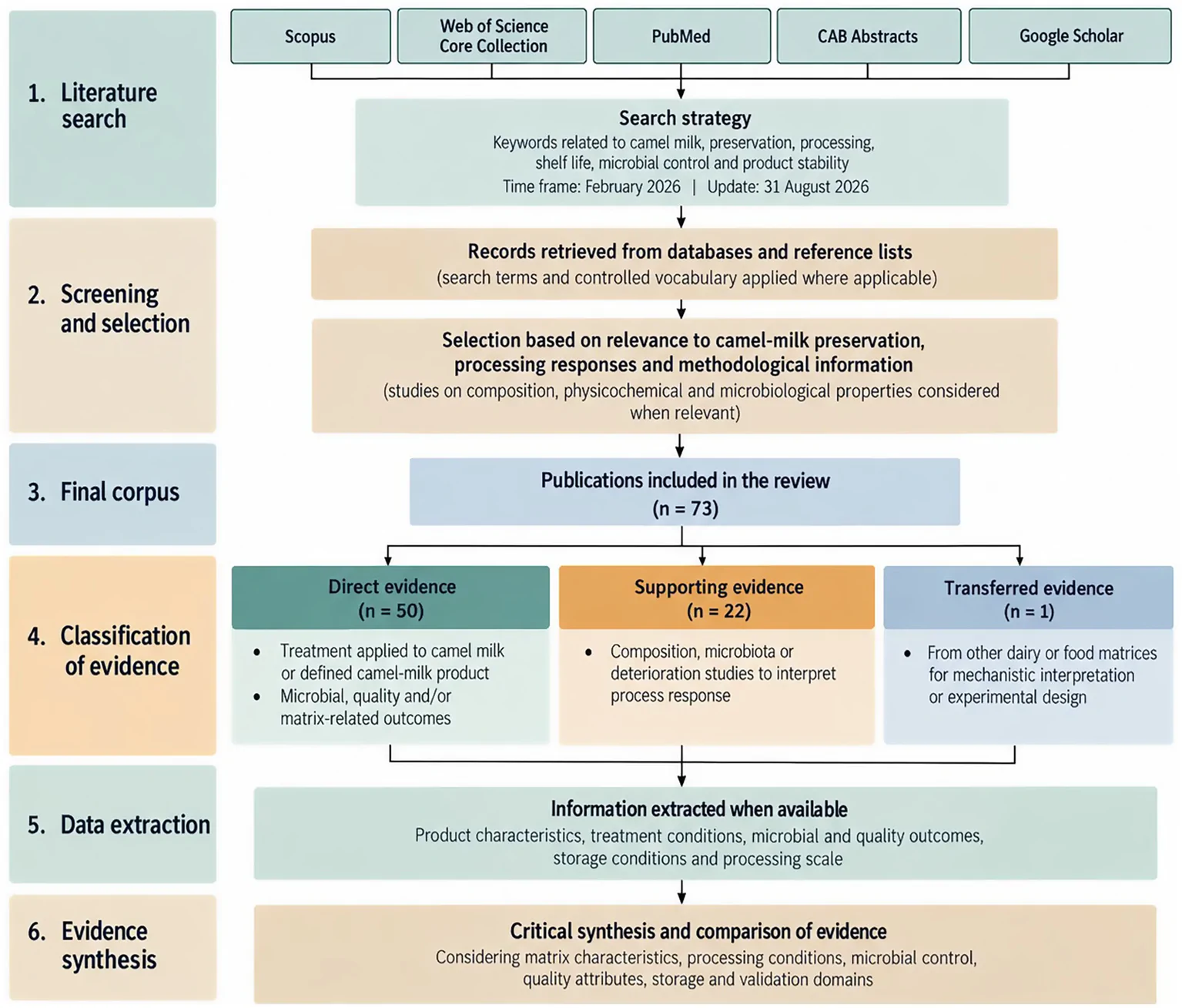
Schematic overview of the literature search, study selection, evidence classification, and critical synthesis approach adopted in this review.

The initial literature search was conducted in February 2026 using Scopus, Web of Science Core Collection, PubMed, and CAB Abstracts, supplemented by Google Scholar. Search combinations covered camel milk, preservation, processing, shelf life, microbial control, and product stability, with database-specific syntax and controlled vocabulary applied where appropriate. Reference lists and citing publications were also examined to identify additional relevant studies. A targeted search update was completed on 31 August 2026.

Studies were eligible for inclusion when they investigated preservation or processing treatments applied to camel milk or defined camel-milk products. Studies addressing compositional, physicochemical, or microbiological properties were also considered when their findings contributed to the interpretation of processing responses. Publications focusing exclusively on animal production, therapeutic or clinical effects, or lacking sufficient methodological information were excluded. Review articles were used to contextualise the literature and identify relevant publications, whereas experimental findings were extracted from original studies.

Evidence was classified according to its relationship with the preservation claims under examination. Direct evidence comprised studies in which a preservation or processing treatment was applied to camel milk or a defined camel-milk product. Supportive evidence included studies on camel-milk composition, microbiota, or deterioration that informed the interpretation of processing responses without directly validating a treatment. Transferred evidence originated from other dairy or food matrices and was considered exclusively for mechanistic interpretation or experimental design. Where available, information was extracted on product characteristics, treatment conditions, microbial and quality outcomes, storage conditions, and processing scale. The final evidence inventory comprised 73 publications, including 50 direct, 22 supportive, and one transferred evidence source. Supplementary Table S1 provides the study-level inventory, specifying camel species, milk or product matrix, process or study design, principal endpoint, evidence category, and contribution to the synthesis.

The evidence was critically synthesised by distinguishing direct experimental findings from mechanistic support and observations transferred from other matrices. Reported microbial efficacy was examined alongside product-quality constraints, storage conditions, processing scale, and packaging. The strength and applicability of preservation claims were assessed according to the experimental design, the extent to which treatment delivery was characterised, the relevance of the measured endpoints, and the availability of evidence supporting process validation. Rather than assigning an overall ranking to preservation technologies, the analysis examined the evidence available across validation domains and identified limitations affecting the interpretation and transferability of reported outcomes.

Differences in camel species and product matrices were explicitly considered when interpreting the findings. Most published processing experiments available in English concern dromedary milk (*Camelus dromedarius*), whereas some studies of naturally fermented milk and milk powders involve Bactrian camels (*Camelus bactrianus*) or identify the source only as “camel milk.” These studies were retained when they provided relevant mechanistic information, but results obtained from Bactrian milk were not used to establish processing conditions for dromedary milk. Species was therefore treated as a source of heterogeneity, alongside geographical origin, diet, production practices, and product characteristics, rather than assuming equivalent processing responses across milk sources.

For each preservation approach, the critical analysis considered the characteristics of the treated milk, the conditions under which the treatment was delivered, the microbial or deterioration endpoints measured, the effects on product quality and functionality, and the extent to which the experimental design supported conclusions beyond laboratory conditions. Three levels of evidence were distinguished: demonstration of a change in microbial counts, achievement of a defined safety or shelf-life target, and reproducibility of the reported performance under industrially relevant conditions. This distinction was maintained throughout the synthesis to avoid interpreting immediate experimental effects as evidence of validated preservation performance.

## 3 Matrix determinants of preservation response

Camel milk is a compositionally heterogeneous matrix whose properties vary with breed, diet, season, water availability, lactation stage, geographical origin and production system. These factors influence total solids, protein and fat contents, mineral balance and initial microbial quality (36–38). Such variability has direct implications for preservation because it modifies heat transfer, buffering capacity, optical attenuation, interfacial behaviour and the availability of substrates for microbial and enzymatic activity. Table 1 summarises the principal matrix attributes affecting treatment delivery, preservation efficacy and product quality, together with their implications for experimental design and interpretation. Figure 2 complements this framework by illustrating the expected sensitivity of different preservation processes to these attributes. Marker size represents the relative strength of matrix influence, whereas colour distinguishes effects primarily associated with treatment delivery or microbial lethality, product quality or functionality, or both.

**Table 1:** Camel-milk matrix attributes that modify preservation-process delivery, efficacy and product quality.

Matrix attribute	Mechanistic consequence	Processes affected	Implication for experimental design and interpretation	References
Between-herd and seasonal variation in fat, protein, solids, pH and minerals	Changes buffering, heat transfer, viscosity, optical attenuation and microbial substrate availability	All processes; especially heat, UV-C, concentration and fermentation	Use independent batches spanning the intended raw-material range; report breed, season, diet and production system	(14, 36–38, 89)
High beta-casein, low kappa-casein and relatively large casein micelles	Weakens micellar surface stabilisation and alters aggregation, rennetability and gel formation	Heating, HPP, concentration, cheese manufacture and drying	Report micelle/mineral context and distinguish aggregation, denaturation and functional gel failure	(21, 23, 24, 26)
Absence of beta-lactoglobulin and distinct whey-protein composition	Changes heat-induced association, deposition and analytical recovery of soluble proteins	Heating, UHT, drying, concentration and pressure	Do not transfer bovine whey markers or denaturation thresholds without camel-milk verification	(27, 40, 42, 53, 54)
Variable calcium, phosphate and ionic equilibria	Controls buffering, colloidal stability, rennet coagulation and heat-induced aggregation	Heating, fermentation, HPP, concentration and cheese manufacture	Report pH and mineral adjustment; separate soluble and colloidal mineral effects	(27, 30, 56, 89)
Fat-globule interfaces and oxidation substrates	Influences cavitation, light scattering, oxygen transfer, creaming and volatile formation	Ultrasound, UV-C, heating, homogenisation and storage	Combine particle-size, oxidation, volatile and sensory endpoints; document oxygen and temperature history	(41, 57, 58, 79)
High optical absorption and scattering in opaque milk	Creates non-uniform fluence and shadowing, especially in static or poorly characterised reactors	UV-C and other optical treatments	Report absorbance/scattering, path length, mixing, residence-time distribution and delivered fluence	(34, 58)
Viscosity, suspended particles and fouling propensity	Changes flow, residence time, acoustic field, heat-transfer coefficients and limiting treatment location	Continuous heating, UV-C, ultrasound, membrane and concentration processes	Characterise flow regime, geometry, fouling and cold- or low-dose locations rather than nominal settings alone	(26, 29, 57, 58)
Endogenous lactoferrin, lactoperoxidase, lysozyme and immunoglobulins	May delay growth or contribute to hurdle effects, but activities vary and are heat sensitive	Cooling, heating, fermentation and lactoperoxidase activation	Measure activity where relevant and avoid treating endogenous inhibition as validated pathogen control	(39, 44, 45, 107)
Variable initial microbiota, spore-formers and psychrotrophic organisms	Determines pre-process enzyme load, spoilage trajectory and apparent treatment response	Cooling, pasteurisation, UHT, fermentation and storage	Document complete time–temperature history and distinguish hygiene improvement from lethality	(11, 17–19)
Udder-health status and somatic-cell-associated enzymes	Can confound raw-milk quality and enzyme-marker interpretation before processing	Raw-milk acceptance, refrigeration and heat-marker studies	Report health indicators or sampling controls when enzyme activity is used as a process marker	(20, 59)
High buffering capacity and culture-dependent acidification	Modifies the time required to reach inhibitory pH and affects gel structure and starter performance	Controlled and spontaneous fermentation	Report culture identity, inoculum, temperature, acidification kinetics, post-acidification and storage	(47, 81, 83, 88)
Lactose-rich amorphous powder phase and moisture sensitivity	Promotes caking, browning, oxidation and loss of flowability when water activity and temperature rise	Spray drying, vacuum drying and powder storage	Report product temperature, water activity, glass-transition behaviour, package barrier and real-time stability	(68, 70, 72, 73, 77)

HPP, high-pressure processing; UHT, ultra-high-temperature treatment; UV-C, ultraviolet-C.

**Figure 2:**
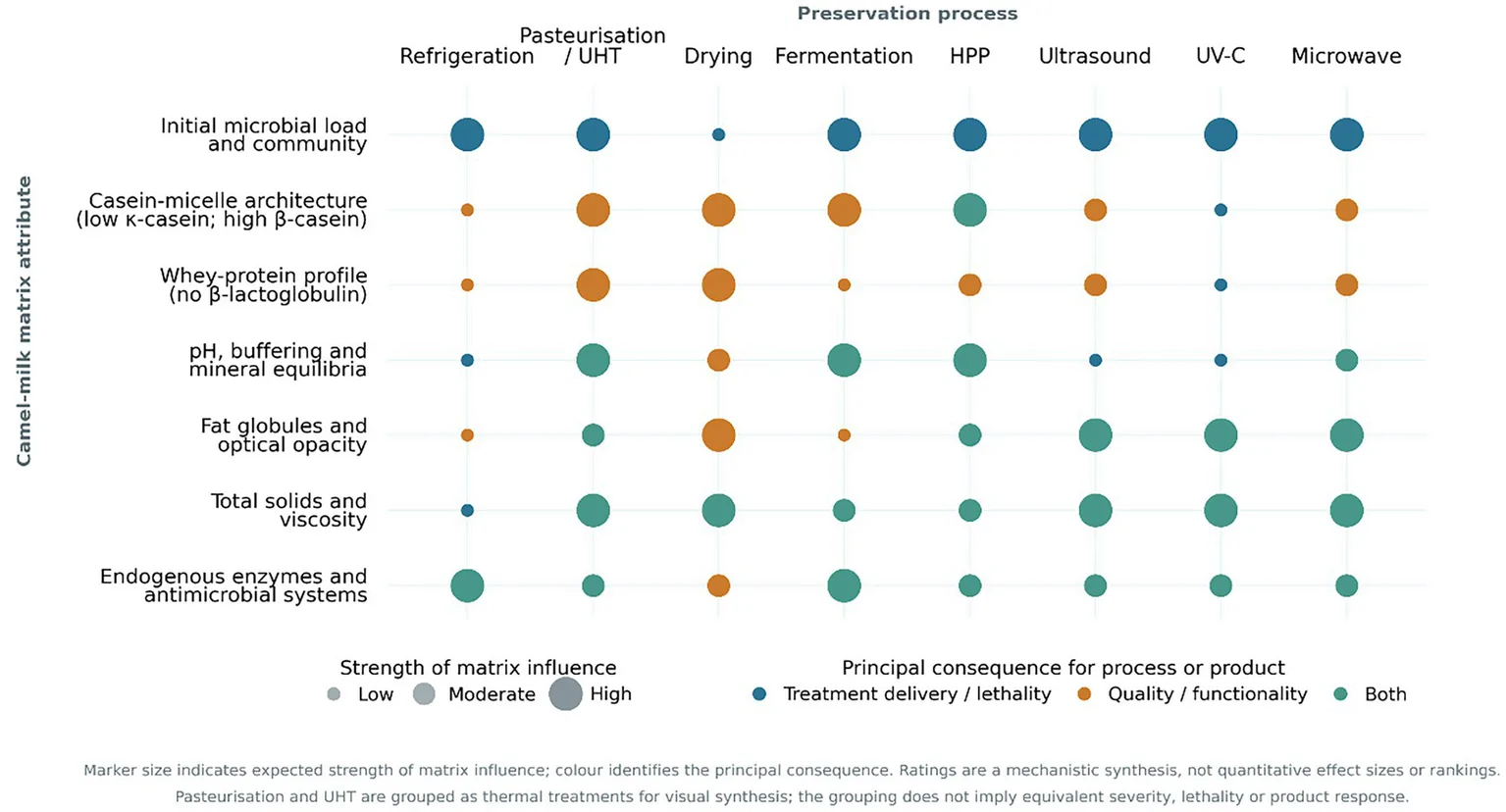
Camel-milk matrix sensitivity across preservation processes.

The relationships presented in Table 1 indicate that camel-milk characteristics influence preservation through multiple, frequently interacting mechanisms. Protein organisation and calcium–phosphate equilibria jointly determine colloidal stability and functional responses to heating, pressure and acidification, whereas fat-globule characteristics and optical properties influence energy distribution and quality changes during ultrasound and UV-C processing. Initial microbiota and endogenous enzyme activity introduce further variability by determining the condition of the milk before treatment and its subsequent deterioration potential. Importantly, these factors cannot be considered independently. Adjustments intended to improve one technological property may compromise another, as illustrated by the contrasting effects of mineral modification on rennet coagulation and heat stability. The combined interpretation of Table 1 and Figure 2 therefore indicates that processing conditions must be evaluated in relation to the matrix characteristics most relevant to the treatment mechanism and intended product, rather than extrapolated directly across different camel-milk systems.

Casein organisation is particularly important for thermal and pressure processing. Compared with bovine milk, camel milk contains a higher proportion of β-casein, substantially less κ-casein and generally larger casein micelles (21, 24). The relatively low abundance of κ-casein may reduce steric stabilisation at the micellar surface, although it does not independently account for the range of instabilities observed during processing. Protein aggregation is also influenced by pH, ionic calcium, colloidal calcium phosphate, total solids, prior homogenisation and thermal history. The markedly shorter heat-coagulation time reported for camel milk under severe heating conditions remains an important indication of its technological sensitivity, but heat stability cannot be defined independently of pH and sample history (26).

The whey-protein fraction introduces additional complexity. Camel milk lacks β-lactoglobulin, while α-lactalbumin, serum albumin, immunoglobulins and lactoferrin contribute to treatment-dependent changes in protein structure and interactions. However, results obtained from purified proteins, acid whey, rennet whey or proteins remaining soluble after heating cannot be considered interchangeable because separation procedures, mineral environments and prior processing influence unfolding, aggregation and analytical recovery. Elagamy (39), Felfoul et al. (40) and Genene et al. (27) demonstrated the importance of evaluating whey-protein responses and rennetability within the relevant milk system. Although the absence of β-lactoglobulin eliminates a major pathway of heat-induced whey protein–κ-casein association characteristic of bovine milk, weak rennet coagulation in camel milk is primarily associated with the organisation of casein micelles, κ-casein availability, calcium equilibrium and processing conditions rather than the absence of this individual whey protein.

The lipid fraction influences preservation through interfacial behaviour, oxidation, creaming and interactions with processing energy. Differences in fat-globule size and fatty-acid composition may affect interfacial area and susceptibility to lipolytic or oxidative deterioration, although comparisons among milk species depend on sampling and analytical methods. Thermal processing can promote volatile formation and Maillard-associated changes, whereas ultrasound may intensify oxidation when cavitation, oxygen incorporation and temperature are insufficiently controlled. Wang et al. (41) demonstrated that volatile profiles and Maillard-reaction products in camel milk vary with thermal severity. Consequently, oxidative stability cannot be established from a single thiobarbituric acid reactive substances (TBARS) measurement; interpretation should also consider volatile compounds, oxygen exposure, packaging and storage duration.

Optical properties represent a particularly important constraint for UV-C processing. Proteins, fat globules and suspended particles absorb and scatter incident radiation, generating heterogeneous fluence distributions within the product. Consequently, the nominal lamp dose does not necessarily represent the exposure received by microorganisms throughout the treated volume. Optical attenuation interacts with reactor path length, mixing, flow regime and lamp fouling, making reactor configuration an integral component of treatment performance. Related considerations apply to ultrasound and microwave processing, for which nominal frequency or equipment power cannot adequately characterise treatment delivery without information on sample volume, geometry, exposure time, temperature history and energy distribution.

Camel milk also contains endogenous antimicrobial components, including lactoferrin, lactoperoxidase, lysozyme-like activity and immunoglobulins (42–45), together with peptides possessing antimicrobial potential. However, antimicrobial activity demonstrated under controlled experimental conditions does not establish effective pathogen control in raw milk. Indigenous microbiota, buffering capacity, substrate availability and environmental conditions can influence the expression and persistence of these effects. Fermentation may generate organic acids and inhibitory peptides, but spontaneous processes remain variable and may allow undesirable microorganisms to persist alongside lactic acid bacteria (46–50). Endogenous antimicrobial systems should therefore be considered potential components of combined preservation strategies rather than substitutes for hygienic production or validated microbial inactivation.

The initial microbial population is itself a determinant of preservation performance because it reflects the conditions prevailing before processing. Raw camel milk may contain mesophilic bacteria, coliforms, staphylococci, spore-forming organisms and microorganisms introduced through milking equipment, water, handlers or the udder (11, 12, 14, 18). Elevated initial contamination can accelerate acidification and the accumulation of extracellular enzymes before treatment. Subsequent microbial inactivation cannot necessarily reverse the deterioration already initiated, particularly when heat-stable proteases and lipases persist after processing. Thus, initial microbial quality and the complete pre-processing temperature history must be considered when interpreting treatment efficacy and subsequent shelf life.

Buffering capacity provides information that cannot be obtained from initial pH alone. Milk samples with similar pH may require different amounts of acid to reach inhibitory conditions because of variations in protein, phosphate and citrate concentrations. These differences influence fermentation kinetics, starter-culture performance and the conditions under which sublethally injured microorganisms may recover. Cooling, heating, pressure treatment and acidification can also modify the distribution of calcium and phosphate between soluble and colloidal phases. Consequently, studies examining protein stability or fermentation should report pH together with titratable acidity or buffering capacity and, where mineral interactions are relevant, distinguish soluble from colloidal mineral fractions.

The physical state of the product further modifies the effects of the milk matrix. Homogenisation changes fat-globule dimensions and interfacial protein adsorption; concentration increases viscosity and molecular interactions; fermentation modifies pH and casein organisation; and drying produces particles with heterogeneous physical structures. Reconstitution may not fully restore the organisation of the original milk. Accordingly, processing responses obtained in raw fluid milk cannot be transferred without qualification to pasteurised milk, fermented products, concentrates, whey fractions or powders.

Published heat-stability investigations illustrate the importance of distinguishing analytical endpoints when interpreting matrix responses. Immunochemical assays, proteomic analyses and soluble-protein measurements do not necessarily describe the same structural event. Loss of antibody recognition may occur before extensive aggregation, whereas reduced recovery from the soluble fraction may reflect protein association or precipitation rather than chemical degradation. Elagamy (39) examined the residual activities of lactoferrin, lactoperoxidase, lysozyme and immunoglobulins after graded heating. Wernery et al. (51) and Lorenzen et al. (52) evaluated indigenous enzyme activities as potential heat-treatment indicators, while Benabdelkamel et al. (53) and Felfoul et al. (40) investigated treatment-dependent changes in whey proteins. Felfoul et al. (54) characterised protein deposition during heating, and Genene et al. (27) examined the relationship between whey-protein denaturation and rennetability. Collectively, these findings demonstrate that thermal responses depend on both the milk system and the analytical endpoint, precluding the definition of a single denaturation threshold applicable to all camel-milk products.

### 3.1 Protein architecture, minerals, and heat-induced transitions

The relative abundance and organisation of individual milk proteins are more informative for processing behaviour than gross casein concentration alone. Kappeler et al. (43, 55) and Mohamed et al. (23) reported a substantially higher β-casein-to-κ-casein ratio in camel milk than in bovine milk, together with the absence of β-lactoglobulin and the presence of whey acidic protein and peptidoglycan-recognition protein. These differences modify the molecular interactions available during thermal processing. In bovine milk, denatured β-lactoglobulin can associate with κ-casein at the micellar surface or remain in the serum phase, depending on pH and mineral conditions. Camel milk lacks this prominent association pathway, yet remains susceptible to heat-induced instability because colloidal behaviour depends on interactions among casein micelles, minerals and serum proteins rather than on any single protein constituent.

Heat stability and rennetability are related but represent distinct technological properties. Farah and Atkins (26) reported short heat-coagulation times under severe thermal conditions and demonstrated the importance of pH. Genene et al. (27) subsequently showed that whey-protein denaturation and rennet coagulation respond differently to heating conditions. Konuspayeva et al. (29) and Kamal et al. (56) investigated processing adjustments, with the latter examining the effects of calcium and phosphate supplementation on coagulation and gel properties in raw and heated milk. These findings illustrate the context-dependent role of mineral equilibrium. Increasing calcium availability may favour rennet-induced aggregation when ionic calcium limits coagulation, but it may also intensify heat-induced protein association under conditions that reduce colloidal stability. Consequently, mineral adjustments that improve cheese manufacture cannot be assumed to enhance the stability of milk intended for UHT processing.

Differences in analytical approaches further complicate comparisons among heat-treatment studies. A reduction in protein recovered from the soluble fraction may indicate aggregation or precipitation rather than irreversible molecular degradation. Similarly, loss of antibody recognition may reflect conformational changes without extensive bulk aggregation, while conventional electrophoresis may not fully characterise cross-linking, oxidation or modifications affecting low-abundance proteins. Benabdelkamel et al. (53) identified treatment-dependent changes in the whey-protein profile, whereas Felfoul et al. (54) characterised protein deposition during heating. These observations address different manifestations of thermal modification and should not be combined into a single estimate of protein denaturation temperature.

Mineral characterisation requires comparable attention to the analytical matrix. Total calcium concentration does not indicate the fraction available for rennet coagulation or heat-induced protein association. Cooling, acidification, concentration and pressure treatment can redistribute calcium and phosphate between soluble and colloidal phases, while pH simultaneously affects mineral solubility and protein charge. Titratable acidity provides complementary information on the amount of acid required to modify the system. Therefore, the processing window relevant to protein stability is multidimensional, and studies reporting only initial pH cannot establish whether differences in buffering capacity or ionic calcium contributed to apparently inconsistent outcomes.

### 3.2 Fat interfaces, oxidation, and optical heterogeneity

The contribution of the lipid fraction to preservation extends beyond its nutritional composition. Homogenisation increases interfacial area and promotes redistribution of caseins and whey proteins onto newly formed fat-globule surfaces. Subsequent heating modifies both these interfacial protein layers and proteins in the continuous phase. Accordingly, homogenisation followed by heating is not necessarily equivalent to heating non-homogenised milk, even when the same bulk temperature is reached. Measurements of mean fat-globule size alone cannot characterise interfacial composition, free-fat formation or the susceptibility of newly generated surfaces to lipolytic and oxidative reactions.

The interpretation of lipid oxidation also depends on the analytical endpoints selected. TBARS measurements primarily reflect a subset of secondary oxidation products and may remain unchanged despite alterations in volatile compounds or lipid composition. Dhahir et al. (57) reported broadly stable fatty-acid profiles, protein fractions and TBARS values following ultrasound treatment, while 18 of the 24 monitored volatile compounds changed. Similarly, Dhahir et al. (58) observed no significant increase in TBARS after UV-C treatment, although conjugated-linoleic-acid isomers shifted and three additional volatile compounds were detected. These findings demonstrate that the absence of a significant response in one oxidation indicator does not establish overall chemical stability. Assessment of treatment-induced deterioration should therefore integrate complementary lipid and volatile endpoints, particularly when flavour retention is relevant to product acceptance.

Optical heterogeneity introduces an additional, process-specific limitation. In UV-C treatment, proteins, fat globules and suspended particles contribute to radiation attenuation through absorption and multiple scattering. Although fat removal may improve optical transmission, it also changes the matrix and therefore the product for which preservation performance is being evaluated. Furthermore, measurements of static absorbance cannot fully characterise the fluence received by microorganisms in continuous-flow reactors, where exposure depends on fluid trajectories and residence-time distributions. Optical properties, reactor path length and hydrodynamic conditions must consequently be considered components of the delivered process rather than merely descriptive characteristics of the milk.

### 3.3 Microbial ecology before treatment

Initial microbial contamination reflects the conditions under which camel milk is produced, collected and transported, and its effects cannot be eliminated simply through statistical adjustment. Udder health, water quality, equipment sanitation, handling practices, delays before cooling and transport temperature influence both the microbial population and the enzymatic condition of milk entering the processing system. Udder-health indicators and subclinical mastitis are particularly relevant because they may alter cell-associated and enzymatic measurements before processing (20, 59). Studies of camel-milk microbiota have identified lactic acid bacteria with potential technological applications alongside staphylococci, Bacillus spp., environmental organisms, psychrotrophs and potentially pathogenic or spoilage-associated microorganisms (11, 18, 19, 60). The presence of potentially beneficial microorganisms does not diminish the safety significance of contaminating populations. Moreover, culture-dependent enumeration and sequencing-based characterisation provide different types of information: sequencing describes community composition, whereas preservation validation requires evidence concerning viable organisms and their potential recovery following treatment-induced injury.

Psychrotrophic microorganisms are particularly relevant when raw camel milk undergoes prolonged holding before pasteurisation. Refrigeration restricts the growth of many mesophilic organisms but permits psychrotrophic populations to persist or proliferate and produce extracellular proteases and lipases. Some of these enzymes may retain activity after subsequent heating, contributing to bitterness, gelation or rancidity despite reductions in viable microbial counts. Consequently, milk processed immediately after collection cannot be considered equivalent to milk subjected to extended warm transportation followed by refrigeration. The complete pre-processing temperature history must therefore be incorporated into the interpretation of preservation efficacy and storage stability.

Spore-forming bacteria introduce a further distinction between the control of vegetative microorganisms and the achievement of long-term product stability. Conventional pasteurisation is not designed to eliminate bacterial spores, whereas UHT and commercial-sterility processes address a more demanding microbiological objective. Nevertheless, published camel-milk studies rarely integrate spore-specific challenge testing, delivered thermal lethality, package integrity and subsequent incubation or storage assessment within the same experimental design. This limitation is particularly consequential for shelf-stable products, because reductions in total microbial counts immediately after processing cannot establish commercial sterility or predict the behaviour of surviving spores during storage.

## 4 Preservation performance across process classes

Preservation technologies are examined within three broad process groups, followed by a comparative assessment of emerging approaches. This organisation emphasises shared processing constraints while distinguishing differences in the nature and maturity of the available evidence. Table 2 summarises representative direct studies of camel-milk preservation and processing, whereas Table 3 identifies the evidence required to progress from an experimentally observed effect to a validated process claim.

**Table 2:** Direct camel-milk preservation and processing studies used to delimit defensible process claims.

Route	Reported processing conditions	Microbial or preservation outcome	Quality or storage outcome	Principal validation constraint	References
Refrigeration	Raw milk, 4 °C, 29 d	Counts increased during prolonged storage; not evidence of 29-d safety	Composition changed with time	No pathogen challenge; initial hygiene differed	(63)
Storage	Raw milk under defined storage conditions	Deterioration depended on time and temperature	Physicochemical quality declined	Does not validate a lethal process	(64)
LTLT	63 °C, 30 min; plate-and-frame	No defined pathogen lethality established	Minor proximate, amino-acid and fatty-acid changes	Nutritional retention is not process validation	(61)
Pasteurisation	Raw versus pasteurised camel milk	Vegetative flora reduced	Physicochemical comparison	Heterogeneous milk history and no standard challenge	(65)
HPP	300–350 MPa; up to 5 min	D-values 1.48–3.77 min; about 2–3 log pathogen reduction; background flora <1 log	No marked proteolysis or micelle deterioration; 4/10 °C storage	*L. monocytogenes* limiting; no pasteurisation equivalence	(62)
Ultrasound	100 mL; several nominal powers	About 4 log background-count reduction at 140 W	Selected proteins/bioactivities followed for 7 d at 4 °C	No defined pathogen challenge; delivered power not reported	(78)
Ultrasound	70 mL; 13-mm probe; 15 min; ice cooling	*E. coli* O157: H7 and *S. typhimurium* challenged	TBARS/proteins broadly stable; 18/24 volatiles changed	Batch geometry and active cooling limit scale transfer	(57)
UV-C	Continuous flow; 4.15–12.45 mJ/cm^2^	At 12.45 mJ/cm^2^: 3.9 log for *E. coli* O157: H7 and *S. typhimurium*	Selected proteins/TBARS stable; CLA isomers shifted; 3 new volatiles	Did not reach authors’ 5-log target; optical dose distribution unresolved	(58)
Microwave	Time–temperature comparative treatment	No microbial lethality established	Protein, structure and micromorphology changed	Requires cold-point mapping and equivalent-lethality comparison	(76)
Spray drying	Increasing inlet temperature	Low-moisture powder, not a lethality validation	Maillard products increased with inlet temperature	Product temperature, aw and storage package govern transfer	(68)
Powder storage	Accelerated storage of spray-dried powder	Microbial growth not the primary endpoint	Physicochemical stability deteriorated	Accelerated conditions require real-time confirmation	(72)
Lactoperoxidase	Activated system at defined storage temperatures	Delayed growth; effectiveness temperature dependent	Temporary holding intervention	Cannot replace pasteurisation	(107)
Packaging	Pasteurised milk in different packages	Package affected shelf life	Barrier properties incompletely reported	Needs OTR, light transmission, seals and abuse testing	(108)
Thermal markers	Raw camel milk; graded heat treatments	ALP, GGT and LPO inactivation examined as process indicators	Enzyme response, not pathogen lethality	Requires validation across raw-milk variability and target lethality	(51)
Mineral/heat	Raw and heated camel milk; calcium and phosphate addition	No microbial endpoint	Rennet coagulation and gel properties changed with heat and minerals	Cheese functionality cannot be transferred to UHT stability	(56)
Fermentation	Low-fat camel milk; 3 cultures; 28, 35 and 42 °C; 96 h	Growth and acidification were culture- and temperature-dependent	pH, acidity, lactose, lactic acid and proteolysis followed	No pathogen challenge; product safety not established	(88)
Heating	Camel, cow and buffalo milk; graded heat treatments	Antimicrobial proteins were treatment-sensitive	Protein activities changed with heat	Does not establish pathogen lethality	(39)
Heating/storage	Camel milk; heat treatments and storage	Shelf-life response depended on treatment	Sensory properties were followed	Limited process-delivery and hazard information	(75)
Fermentation	Whole camel and cow milk; yogurt starters	Associative growth and proteolysis differed by milk	Product-development evidence	No pathogen challenge or industrial validation	(81)
Fermentation	Dromedary milk; lactic fermentation	Microbiological acidification response characterised	Rheological development measured	Laboratory product study	(47)
Yogurt	Set-type camel-milk yogurt	No safety target evaluated	Quality and acceptability assessed	Formulation evidence, not preservation validation	(85)
Spray drying	Whole camel milk powder; process optimisation	No microbial lethality target	Drying parameters and powder production assessed	No challenge test or long-term package validation	(77)
Ultrasound	Moroccan camel milk; controlled ultrasound for 5–15 min	Approximately 2-log reduction after 5 min; bacteria not detected after 15 min under the reported recovery conditions	Reduced particle size and turbidity; altered zeta potential and protein secondary structure; emulsifying capacity retained	Background flora rather than a defined pathogen challenge; no scale-independent acoustic dose	(79)
Ultrasound-assisted fermentation	Camel, goat and cow milks; ultrasound before, after or before and after fermentation	No pathogen-lethality claim established	Matrix-dependent changes in pH, conductivity, soluble solids, colour and mineral-related multivariate patterns	Physicochemical evidence; microbial safety and storage validation remain untested	(89)

**Table 3:** Minimum evidence and reporting framework for validation of camel-milk preservation processes.

Validation domain	Minimum evidence or reporting	Inference failure prevented	Supporting references
Product identity and intended use	Species, milk state, pretreatment, formulation, package, intended storage and consumer/use scenario	Applying a result from raw, pasteurised, fermented or powdered milk to a different product	(66, 67, 109, 110)
Hazard definition and challenge design	Target pathogen or justified surrogate, strain set, inoculum preparation, inoculation route and physiological state	Using indigenous counts as proof of control of a defined pathogen	(62, 66, 109, 110)
Raw-material representativeness	Independent camel-milk batches; origin, composition, pH, fat, solids, minerals and initial microbiota	Confusing analytical triplicates with biological replication or assuming one pooled batch is universal	(19, 36, 38, 62)
Delivered treatment and dose distribution	Time–temperature, pressure, fluence or specific energy measured in the product; geometry, volume, flow and limiting location	Equating nominal power, lamp output, pressure setting or bath temperature with delivered treatment	(57, 58, 66, 67, 78, 79, 105)
Temperature history and process interactions	Come-up and cooling times, adiabatic or acoustic heating, holding profile and pretreatments	Attributing a combined thermal/non-thermal effect to one mechanism or overlooking cumulative lethality	(57, 62, 110)
Enumeration, injury and uncertainty	Independent process replicates, detection and quantification limits, confidence intervals and recovery of injured cells	Reporting below-detection values as complete inactivation or treating *p*-values as process equivalence	(58, 62, 109, 110)
Storage validation and failure criterion	Relevant duration and temperature distribution, post-process recovery, spoilage/pathogen limit and predeclared failure criterion	Extrapolating an immediate reduction to shelf life or commercial sterility	(63, 64, 72, 109)
Quality, nutrition and functionality boundary	Prespecified acceptable limits for aggregation, sediment, oxidation, volatiles, flavour, gelation and key bioactivities	Selecting a microbiologically effective condition that yields an unacceptable product	(27, 41, 57, 58, 78)
Scale-up, fouling and routine verification	Pilot or continuous operation, residence-time distribution, fouling, process marker, monitoring and corrective action	Promoting a small static-batch effect as an industrially controllable process	(51, 52, 66, 67, 111)
Package and distribution integration	Hygienic filling, seal integrity, oxygen/light/moisture barrier, abuse conditions and cold-chain performance	Losing the treatment benefit through recontamination or an unsuitable package	(33, 66, 72, 108)
Economic and environmental feasibility	Throughput, energy and water demand, cleaning, yield loss, refrigerant/cold-chain dependence and waste	Recommending technically effective conditions that cannot be sustained in the target production chain	(1–3, 32)

The studies assembled in Table 2 reveal an important distinction between the outcomes measured and the conclusions those outcomes can support. Investigations of thermal processing, mineral adjustment and drying frequently provide detailed information on nutritional retention, protein functionality or physicochemical stability without establishing lethality against defined microbial hazards. Conversely, studies incorporating microbial challenges may provide stronger evidence of pathogen inactivation while leaving treatment uniformity, long-term product stability or industrial reproducibility unresolved. This contrast is illustrated by the nutritional assessment of industrial low-temperature, long-time (LTLT) pasteurisation by Arain et al. (61), the pathogen-specific high-pressure processing (HPP) investigation by Osaili et al. (62), and the continuous-flow UV-C study by Dhahir et al. (58). Although all three contribute directly to understanding camel-milk preservation, they address different questions and cannot be ranked using a common efficacy measure. Table 2 should therefore be interpreted as a comparison of experimentally supported outcomes and their limitations rather than as a hierarchy of preservation technologies.

### 4.1 Cold, thermal and dehydration routes

Rapid cooling remains among the most accessible interventions for preserving camel milk after milking, but refrigeration delays microbial growth rather than providing a lethal treatment. Although lower temperatures restrict the proliferation of many mesophilic microorganisms, psychrotrophic populations and their extracellular enzymes may remain active during storage. Consequently, the effectiveness of refrigeration depends on initial hygiene, the delay before cooling and the subsequent temperature history. Farid et al. (63) monitored raw camel milk at 4 °C for 29 days and documented changes in composition and increasing aerobic bacterial counts. These findings characterise deterioration during prolonged refrigeration but do not establish a safe 29-day shelf life. Similarly, Wang et al. (64) demonstrated that storage conditions influence raw-milk quality, reinforcing the need to distinguish the effects of storage temperature from those of initial contamination and pre-refrigeration handling.

Pasteurisation has a stronger practical foundation for fluid camel milk, although published studies differ considerably in processing conditions, microbial targets and quality endpoints. LTLT treatments can reduce vegetative microbial populations while producing relatively limited immediate changes in selected compositional attributes. However, increasing thermal severity may promote whey-protein denaturation, aggregation, cooked flavours and changes in volatile composition (39, 61, 65). The microbial efficacy of a thermal process cannot be inferred from its nominal temperature alone, because heating and cooling profiles, holding-time accuracy and post-process contamination influence the outcome (66, 67). Moreover, reductions in indigenous microbial counts do not substitute for validation against relevant pathogens. Verification is further complicated by the uncertain suitability of endogenous enzyme markers established for bovine milk when applied to camel milk (51, 52).

UHT processing offers opportunities for ambient distribution but introduces substantial challenges related to the stability of the camel-milk matrix. Severe heating can promote protein aggregation, sedimentation, Maillard reactions and undesirable flavour development (26, 27, 41). Adjustments to pH, mineral equilibrium or stabilising salts may improve selected processing characteristics, but their effects must be assessed in relation to product identity, regulatory requirements and long-term stability. Commercial sterility should not be equated with the elimination of every bacterial spore; it is established through a validated integrated process, appropriate hermetic packaging and defined storage conditions (66, 67). Accordingly, the technological challenge extends beyond achieving sufficient thermal lethality to maintaining colloidal stability and product acceptability throughout storage.

Dehydration introduces a different preservation mechanism by reducing water availability for microbial growth, while simultaneously exposing milk constituents to thermal and physical stresses. Spray drying is industrially scalable, but inlet temperature alone does not adequately describe the thermal history experienced by particles, which also depends on feed concentration, droplet size, airflow and outlet conditions. Perusko et al. (68) reported increased formation of Maillard-reaction products at higher spray-drying inlet temperatures. Al-Thaibani et al. (69) demonstrated that spray drying and ultrasonication influence camel-whey-powder solubility, crystallinity and emulsifying properties, while Kang et al. (70) identified differences in microstructure and protein profiles between spray drying and low-temperature vacuum drying. Although gentler drying conditions may improve retention of selected quality attributes, energy consumption, throughput and reconstitution performance remain essential considerations. Freeze drying provides a useful quality comparator but is not necessarily economically equivalent to industrial spray drying. Regardless of the dehydration method, moisture sorption, lactose crystallisation, caking, oxidation and packaging barriers determine subsequent powder stability (71–74).

The evidence summarised in Table 2 also illustrates why refrigeration, thermal processing and drying cannot be evaluated using identical preservation endpoints. Refrigeration studies primarily describe changes in microbial populations and quality during storage, whereas pasteurisation studies may examine microbial reduction, nutrient retention or protein functionality. Drying investigations frequently focus on powder characteristics and physicochemical deterioration rather than defined microbial lethality. These differences do not diminish the relevance of the individual studies, but they restrict the conclusions that can be drawn from direct comparisons. In particular, favourable compositional or functional outcomes cannot independently establish microbial safety, while an effective microbial treatment does not necessarily ensure acceptable product stability.

Cold-chain performance must be interpreted in relation to the complete temperature history. Microorganisms can proliferate and produce extracellular enzymes during transport before milk reaches refrigeration temperature, and subsequent cooling does not reverse these changes (18, 63). Temperature fluctuations during storage further complicate interpretation relative to experiments conducted under constant laboratory conditions (64). Psychrotrophic populations may also produce heat-stable proteases and lipases that contribute to deterioration after pasteurisation, even when viable microbial counts have been reduced. Consequently, shelf-life assessment requires information on the delay before cooling, subsequent temperature exposure, microbial behaviour, enzymatic deterioration and product-quality failure.

Comparisons among thermal technologies require equivalent microbial lethality rather than reliance on nominal processing temperature or immediate product acceptance. LTLT, high-temperature, short-time (HTST), extended high-temperature treatments and microwave heating may generate different cumulative thermal effects despite similar maximum temperatures (66, 67). Prolonged heating and cooling phases can increase cumulative exposure, whereas rapid heating may generate spatial temperature heterogeneity and leave portions of the product insufficiently treated. Meaningful comparisons therefore require integrated time–temperature histories, relevant microbial inactivation kinetics and an assessment of treatment uniformity.

The verification of thermal processing remains a further limitation. Endogenous enzymes used as pasteurisation indicators in bovine milk cannot automatically be considered suitable for camel milk. A useful indicator must be consistently detectable in raw milk, exhibit an inactivation response appropriately related to the target treatment and remain sufficiently reproducible across variations in milk composition and production conditions (51, 52). Until such relationships are established, monitoring the delivered thermal process remains essential.

Powder studies similarly require evaluation beyond immediate composition. Water activity, moisture content, glass-transition behaviour and packaging conditions determine whether powders remain physically stable or undergo caking, browning and oxidative deterioration during storage (68, 72, 73). Reconstitution performance provides additional information that cannot be inferred from total protein concentration alone, because denaturation and aggregation may reduce solubility without substantially changing bulk composition.

#### 4.1.1 Microbial efficacy and quality responses to thermal processing

Direct thermal investigations differ substantially in their experimental objectives and therefore in the strength of the conclusions they support. Arain et al. (61) evaluated camel milk processed at 63 °C for 30 min in an industrial plate-and-frame system and reported relatively minor changes in proximate composition, amino acids and fatty-acid profiles. These findings support retention of the measured nutritional attributes under the tested conditions but do not establish pathogen-specific lethality. Elagamy (39), Lund et al. (75) and Chethouna et al. (65) provide complementary information on microbial, physicochemical and sensory responses to heating. As indicated in Table 2, differences in raw-milk characteristics, treatment conditions and measured endpoints prevent these findings from being combined into a single estimate of pasteurisation efficacy.

The available evidence supports the conclusion that low-temperature pasteurisation can reduce vegetative microbial populations while retaining much of the measured bulk composition, although the magnitude of microbial control and subsequent shelf-life extension remains dependent on the process and initial milk quality. Protein functionality may deteriorate even when total protein content remains relatively unchanged. Benabdelkamel et al. (53) identified treatment-dependent modifications in whey-protein profiles, Felfoul et al. (54) characterised protein deposition during heating, and Wang et al. (41) demonstrated changes in Maillard-reaction products and volatile compounds with increasing thermal severity. These findings are not necessarily contradictory: preservation of proximate composition can coexist with molecular modifications that affect functionality, flavour and storage stability. Consequently, thermal quality assessment should incorporate endpoints capable of detecting changes relevant to the intended product rather than relying exclusively on bulk nutritional composition.

The interpretation of endogenous enzyme activity as a thermal-processing indicator requires similar caution. Elagamy (39) investigated changes in antimicrobial-protein activity after heating, while Wernery et al. (51) examined alkaline phosphatase, γ-glutamyl transferase and lactoperoxidase as potential indicators of heat treatment. Lorenzen et al. (52) further evaluated indigenous enzyme activities in raw and pasteurised camel milk. The suitability of these markers depends on their initial activity, variability among milk sources, analytical sensitivity and relationship to microbial inactivation. A marker that is inconsistently detectable or inactivates under conditions unrelated to the intended microbial target cannot reliably verify process adequacy. Direct monitoring of treatment conditions therefore remains necessary until marker performance has been validated in representative camel-milk systems. Heat treatment can alter milk oligosaccharide profiles, with effects differing across mammalian milks (114).

#### 4.1.2 UHT, commercial sterility and storage failure

Published evidence supporting the industrial validation of UHT camel milk remains limited relative to its commercial availability. Commercial production does not necessarily imply that detailed challenge studies, residence-time distributions or long-term stability data have been reported in the scientific literature. Laboratory heating experiments provide valuable mechanistic information, but they cannot independently establish the performance of a complete industrial process. Commercial sterility depends on the combined effectiveness of thermal treatment, hermetic packaging and subsequent storage conditions rather than the absolute destruction of every bacterial spore (66, 67).

Three principal failure mechanisms must be distinguished. Insufficient microbial lethality may permit survival or subsequent recovery of spores or injured cells. Heat-induced protein aggregation can generate immediate flocculation or progressive sedimentation during storage. In addition, residual enzymatic activity may contribute to delayed destabilisation even when microbiological criteria have been satisfied (26, 27, 41). These mechanisms respond differently to pH adjustment, mineral modification, homogenisation, preheating and storage temperature. Consequently, acceptable appearance immediately after processing cannot be considered sufficient evidence of long-term UHT stability.

The comparison of UHT with other thermal approaches must account for the complete time–temperature profile. Microwave heating, conventional batch processing and continuous indirect heating may reach similar nominal maximum temperatures while differing in heating rates, residence-time distributions and cooling histories. Zhang et al. (76) demonstrated treatment-dependent changes in camel-milk protein composition, structure and microstructure during microwave heating but did not establish equivalent microbial lethality. These results therefore provide direct physicochemical evidence rather than proof of preservation efficacy. Establishing a meaningful comparison requires identification of the least-treated product fraction and assessment of microbial inactivation under equivalent thermal conditions. Drying method also affects the microstructure and composition of milk fat globules (115).

#### 4.1.3 Dehydration and the transition from process to storage

Drying inhibits microbial growth primarily through reduction of water activity, but the thermal and physical changes occurring during dehydration influence subsequent powder stability. Aralbayev et al. (77) investigated operating conditions for spray drying, whereas Perusko et al. (68) reported increased Maillard-reaction-product formation at higher inlet temperatures. Ho et al. (72) demonstrated continuing physicochemical changes during accelerated storage of spray-dried camel-milk powder, while Kang et al. (70) compared the microstructure and protein profiles resulting from spray drying and low-temperature vacuum drying. Considered together, these studies distinguish the effects of powder formation from deterioration occurring after packaging. This distinction is central to interpreting the evidence in Table 2, because favourable processing outcomes do not necessarily predict long-term storage stability.

Moisture content alone is insufficient to characterise powder stability. Water activity, amorphous lactose, glass-transition behaviour, package permeability and storage temperature determine the susceptibility of particles to caking, browning and other physical changes. Reconstitution must also be assessed through complementary measures, including wettability, dispersibility, dissolution, sedimentation and protein solubility. These properties may respond differently to processing, and total protein content cannot adequately describe their deterioration. Freeze drying offers a useful low-temperature reference, although differences in processing time, energy requirements and throughput limit direct economic comparisons with spray drying.

The evidence also highlights the importance of product identity and species when interpreting drying performance. Whole milk, skim milk, whey concentrates and isolated whey powders differ in fat content, protein composition, interfacial characteristics and prior processing history. These differences affect oxidation, moisture interactions, particle formation and reconstitution. Similarly, findings obtained from different camel species or incompletely characterised milk sources cannot automatically define a common operating window. As documented in Supplementary Table S1, these distinctions must be retained when evaluating the transferability of drying conditions and product-quality outcomes.

### 4.2 Pressure, acoustic, optical and electromagnetic routes

High-pressure processing currently provides some of the most developed pathogen-specific evidence among emerging camel-milk preservation technologies, although the conditions most extensively investigated do not establish pasteurisation equivalence. Osaili et al. (62) reported D-values of 3.77 ± 0.36 min for *Listeria monocytogenes* and 2.10 ± 0.13 min for Salmonella spp. at 350 MPa, and 1.48 ± 0.08 min for *Escherichia coli* O157: H7 at 300 MPa. Treatments lasting up to 5 min produced approximately 2–3 log reductions in the challenged pathogens, whereas total aerobic bacteria, yeasts and moulds, and lactic acid bacteria declined by less than 1 log at 350 MPa. No marked deterioration of casein-micelle structure or protein integrity was observed under the tested treatment and storage conditions. These findings identify a processing region compatible with retention of selected quality attributes, but also identify *L. monocytogenes* as the most resistant organism under the evaluated treatment conditions. The divergence between pathogen-specific inactivation and background microbial reductions further indicates that indigenous flora cannot be used as a general surrogate for pathogen lethality. Industrial application would require further investigation of pressure–time combinations or combined hurdles, recovery of sublethally injured cells, refrigerated shelf-life performance and packaging integrity. Pasteurization and high-pressure processing have also been compared for camel and bovine cheese quality (113).

Ultrasound has demonstrated microbial reduction and modifications of camel-milk structure, but comparison among studies remains constrained by incomplete characterisation of acoustic treatment delivery. Mudgil et al. (78) treated 100-mL portions and reported approximately 4-log reductions in background microbial counts at 140 W, with counts below detection at higher nominal power settings and retention of selected protein and bioactivity measures during seven days at 4 °C. Dhahir et al. (57) investigated *E. coli* O157: H7 and *Salmonella Typhimurium* in pasteurised camel milk using a 13-mm probe applied to 70-mL samples for 15 min under ice cooling. More recently, El Kindoussy et al. (79) reported an approximately 2-log reduction after 5 min and no detectable bacteria after 15 min under the reported recovery conditions. Treatment also reduced particle size and turbidity, modified zeta potential and protein secondary structure, and preserved the measured emulsifying capacity. Collectively, these findings establish microbial and physicochemical responses under the respective experimental conditions, but do not provide a scale-independent acoustic dose or demonstrate pasteurisation equivalence. Calorimetric power, specific energy, amplitude, probe geometry, dissolved gas and the complete temperature history remain essential parameters for interpreting and comparing ultrasound treatments.

UV-C treatment provides quantitative evidence of pathogen inactivation in a continuous-flow camel-milk system. Dhahir et al. (58) evaluated doses of 4.15, 8.30 and 12.45 mJ/cm^2^ in pasteurised milk inoculated with *E. coli* O157: H7 and *S. typhimurium*. The corresponding reductions were 1.9, 3.3 and 3.9 log for *E. coli* O157: H7 and 0.9, 3.0 and 3.9 log for *S. typhimurium*. The highest evaluated dose remained below the 5-log target adopted by the authors. Although TBARS and selected protein profiles were not significantly altered, conjugated-linoleic-acid isomers shifted and three additional volatile compounds were detected. These results demonstrate the importance of assessing microbial efficacy and chemical quality simultaneously. Because camel milk is optically heterogeneous, attenuation and scattering can produce uneven fluence distributions, particularly when reactor hydrodynamics and residence-time variability are considered. Therefore, reactor path length, flow conditions, number of passes, lamp ageing, fouling and characterisation of delivered fluence are integral to interpreting treatment efficacy and its potential transfer to other configurations.

Microwave processing is considered a rapid thermal approach rather than evidence of a distinct non-thermal antimicrobial mechanism. Zhang et al. (76) documented time–temperature-dependent changes in camel-milk protein composition, structure and microstructure but did not establish microbial lethality. Consequently, preservation claims require spatial temperature characterisation and validation at the least-heated location. Gamma irradiation has been investigated in camel whey powder (80), where penetration may offer processing advantages, although product-specific relevance, regulatory requirements and retention of bioactive constituents constrain extrapolation to fluid milk. Pulsed electric fields (PEF) and cold plasma remain prospective research approaches in the context of the evidence reviewed. Without direct camel-milk investigations reporting relevant treatment parameters, microbial outcomes and product-quality responses, their preservation efficacy and industrial suitability cannot be established. Their inclusion in the evidence framework identifies research opportunities rather than demonstrated technological readiness. Recent work on ozone treatment in milk highlights the limitations of extrapolating from aqueous disinfection models to dairy processing (112).

### 4.3 Comparative interpretation of emerging processes

The findings presented in Table 2 demonstrate why emerging camel-milk preservation technologies cannot be ranked solely according to reported microbial reductions. Osaili et al. (62) investigated defined pathogens and estimated organism-specific D-values, providing stronger evidence for hazard-specific inactivation than studies relying exclusively on indigenous microbial counts. Mudgil et al. (78) and El Kindoussy et al. (79) examined background microbial populations alongside selected physicochemical or structural characteristics, contributing valuable information on treatment-induced matrix responses but not establishing pathogen-specific lethality. Dhahir et al. (57, 58) employed inoculated pathogens in ultrasound and UV-C experiments, respectively, although the treatment configurations differed substantially. A reported reduction of 3.9 log in a continuous-flow UV-C system cannot therefore be directly compared with a reduction in indigenous flora following batch ultrasound without accounting for the target organism, initial contamination, recovery conditions, treatment temperature and analytical detection limits.

Interpretation of HPP inactivation kinetics also depends on the assumptions underlying the fitted survival model. D-values are meaningful within the pressure, temperature and exposure conditions under which the relevant portion of the survival curve is adequately represented by log-linear behaviour. Shoulders, tails and differences in strain resistance can limit extrapolation beyond the observed experimental range. Accordingly, the D-values reported by Osaili et al. (62) must be interpreted together with their uncertainty and treatment conditions rather than converted into universal processing times. The relatively small reduction of background microbial populations at 350 MPa further demonstrates that indigenous flora and inoculated pathogens may respond differently to the same treatment.

For ultrasound, the principal limitation concerns the relationship between nominal equipment settings and the acoustic energy delivered to the milk. Generator power does not necessarily correspond to the energy effectively transferred to the sample because losses occur within the transducer and coupling system. Calorimetry estimates energy transferred as heat, whereas acoustic-field measurements provide complementary information on cavitation-related exposure. Probe diameter, immersion depth, treatment volume, dissolved gases and cooling conditions influence the distribution of acoustic intensity. Consequently, the results reported by Mudgil et al. (78), Dhahir et al. (57) and El Kindoussy et al. (79) cannot be reduced to a common power–time relationship. Their principal contribution is to establish treatment responses within defined experimental configurations while identifying the parameters required for subsequent scale-up studies.

The transferability of UV-C findings is similarly dependent on reactor configuration. Dhahir et al. (58) reported progressively greater pathogen reductions with increasing nominal dose, but convergence towards 3.9 log at the highest tested condition does not establish uniform fluence throughout the treated milk. Differences in optical attenuation, flow behaviour, lamp fouling and residence-time distribution may alter the fraction receiving the lowest exposure. The reported reductions therefore characterise the tested continuous-flow configuration rather than a universally applicable UV-C dose for camel milk.

Considered together, the emerging-process studies reveal an important distinction between the magnitude of an observed microbial reduction and the strength of the evidence supporting its application. HPP provides organism-specific inactivation kinetics, but the investigated operating conditions remain insufficient for a general pasteurisation-equivalence claim. Ultrasound studies demonstrate microbial and structural responses, although differences in acoustic delivery, treatment geometry and microbial endpoints limit comparisons across experiments. UV-C provides quantitative pathogen-reduction data under continuous flow, but its performance remains dependent on optical attenuation and reactor hydrodynamics. The most informative distinction among these technologies is therefore not which produces the greatest reported reduction, but which aspects of microbial efficacy, treatment delivery and product stability have been established and which remain unresolved. This interpretation reinforces the need for comparative studies conducted with equivalent microbial targets, defined treatment delivery and common product-quality endpoints.

### 4.4 Fermentation, endogenous systems, packaging, and hurdle designs

Controlled fermentation serves both as a preservation strategy and as a means of transforming camel milk into products with distinct sensory and technological characteristics. Starter-culture performance and gel development depend on microbial strains, milk composition, formulation and processing conditions (81–87). Defined starter cultures can promote more predictable acidification and may generate inhibitory compounds, whereas spontaneous fermentation is influenced by indigenous microbiota, environmental conditions and traditional back-slopping practices. However, reproducible acidification or the production of antimicrobial metabolites does not independently demonstrate control of relevant pathogens. Fermentation must therefore be evaluated in relation to microbial safety, product quality and behaviour throughout storage.

Experimental findings illustrate the importance of distinguishing fermentation performance from preservation validation. Chelladhurai et al. (88) compared low-fat camel and bovine milk fermented with *Lactobacillus helveticus*, *Lactobacillus delbrueckii* subsp. bulgaricus and *Streptococcus thermophilus* at 28, 35 and 42 °C for 96 h. Culture and temperature influenced microbial growth, acidification, lactose utilisation, lactic-acid formation and proteolysis, while milk type affected several responses. Although relationships between titratable acidity and pH suggested similarities in overall buffering behaviour, microbial growth and proteolytic activity were not equivalent across the two milk matrices. Boukria et al. (89) further demonstrated matrix-dependent physicochemical and mineral-related responses when ultrasound was applied before, after or both before and after fermentation. These findings show that culture selection and processing sequence cannot be optimised using acidification time alone, particularly when product structure, microbial safety and storage stability are also relevant.

The technological limitations of fermented camel-milk products extend to gel formation and texture. Studies involving stabilisers, mixed-milk formulations and processing adjustments have demonstrated the sensitivity of rheological and sensory properties to formulation and treatment conditions (90–92). Weak gel formation, syneresis and phase separation may compromise product acceptability and alter the local physicochemical environment. Adjustments involving starter selection, solids concentration, hydrocolloids or mineral supplementation can improve selected quality attributes but may simultaneously influence buffering capacity, diffusion and microbial behaviour. Marete et al. (87) demonstrated the importance of fermentation time and stabiliser formulation for camel-milk yogurt properties, although optimisation of texture or viscosity cannot substitute for microbial challenge testing or storage validation.

Traditional fermented camel milks provide valuable sources of microbial diversity and potential starter cultures, but their microbiological composition varies across products and production environments. Studies of gariss, suusac, shubat and related fermented milks have identified diverse lactic acid bacteria and yeasts, with community composition influenced by geographical origin, processing practices and analytical methodology (18, 48, 50, 93, 94). Additional investigations of traditional products and associated microorganisms have highlighted opportunities for selecting cultures with desirable technological characteristics (49, 84). However, microbial diversity and the presence of potentially beneficial organisms do not establish consistent pathogen exclusion during spontaneous fermentation. Standardisation requires defined cultures, reproducible acidification and verification of microbial behaviour under the intended production and storage conditions.

The identification of antimicrobial peptides and bacteriocin-producing microorganisms offers further opportunities for controlled fermentation and combined preservation strategies. Bioactive peptides derived from camel-milk proteins have demonstrated antimicrobial or other biological activities under experimental conditions (95–102). Lü et al. (103) and Rahmeh et al. (104) investigated antimicrobial compounds produced by microorganisms associated with camel milk, while Algboory and Muhialdin (46) demonstrated antimicrobial peptide activity following fermentation with *Lactobacillus plantarum* IS10. Nevertheless, activity observed in purified fractions, hydrolysates or model systems cannot be directly translated into whole-product preservation efficacy. Peptide concentration, degradation, interactions with casein and fat, and sensory compatibility may influence activity in the finished product. Appropriate validation therefore requires assessment of the complete food system rather than reliance on isolated antimicrobial assays.

Three distinct levels of evidence should be considered when evaluating fermented camel-milk preservation. Acidification kinetics establish how rapidly the matrix reaches conditions potentially inhibitory to microbial growth. Storage investigations determine whether undesirable microorganisms, post-acidification, yeast or mould proliferation and physical deterioration compromise product stability. Pathogen challenge studies establish whether the finished product controls a defined organism under the intended conditions. The findings of Chelladhurai et al. (88) primarily inform fermentation kinetics and associated biochemical responses, whereas antimicrobial peptide investigations such as Algboory and Muhialdin (46) provide complementary mechanistic evidence. Neither type of experiment independently establishes the safety or shelf life of a finished fermented product.

Endogenous antimicrobial systems may contribute to preservation when used as part of a broader hurdle strategy. Activation of the lactoperoxidase system has been shown to delay microbial growth under defined conditions, with effectiveness influenced by storage temperature and substrate availability (105–107). However, the system does not eliminate contamination and its effectiveness diminishes when storage conditions exceed the period or temperature range under which inhibition has been demonstrated. Its potential application is therefore as a temporary holding intervention, subject to applicable national and Codex provisions, rather than as a replacement for pasteurisation or another validated lethal treatment. Similar considerations apply to bacteriocins, essential oils and antimicrobial nanoemulsions, whose use requires evidence of activity in the intended matrix, sensory compatibility and regulatory acceptability.

Packaging represents a further determinant of whether the benefits of preservation are maintained during distribution. Hygienic filling limits recontamination, while oxygen and light barriers can reduce oxidative deterioration and moisture barriers are particularly important for powders. Mohamed Abdalla (108) reported that packaging material influenced the shelf life of pasteurised camel milk, but incomplete information on oxygen and light transmission, headspace conditions, seal integrity and temperature abuse limits generalisation. Active packaging systems incorporating lactoferrin or plant-derived antimicrobials remain of interest, although inhibition demonstrated in isolated packaging materials cannot establish product-level efficacy without considering migration, stability, sensory effects and regulatory requirements. The appropriate packaging system must therefore be evaluated in relation to the processed product and its intended storage conditions.

The evidence assembled in Table 2 highlights that fermentation, endogenous antimicrobial systems and packaging act through different preservation mechanisms and consequently require distinct validation endpoints. Controlled fermentation modifies the product through microbial activity and acidification, whereas lactoperoxidase activation primarily delays microbial growth under specified conditions. Packaging contributes to maintaining the effects of processing by limiting recontamination or deterioration during storage. These approaches may be complementary, but none can be considered a universally sufficient preservation intervention. Their performance must be established within the complete product–process–storage system rather than inferred from isolated antimicrobial, physicochemical or packaging measurements.

Combined hurdle strategies offer opportunities to address the specific constraints of camel-milk production and distribution. Hygienic collection, rapid cooling and mild pasteurisation may be appropriate for refrigerated fluid milk, whereas controlled fermentation combined with temperature management may offer advantages in decentralised production systems. HPP or engineered non-thermal treatments may provide additional options for selected packaged products where process monitoring and refrigerated distribution can be maintained. The objective is not to maximise the number or intensity of treatments, but to combine complementary barriers against relevant microbial and quality-related failure mechanisms while minimising cumulative deterioration. The effectiveness of such combinations must ultimately be demonstrated for the intended product, packaging system and distribution conditions.

## 5 From laboratory efficacy to process validation

A central limitation of the available evidence on camel-milk preservation is the incomplete progression from experimental efficacy to validated process performance. Demonstrating microbial reduction or retention of selected quality attributes under controlled laboratory conditions does not necessarily establish that a treatment will consistently achieve a defined safety or shelf-life objective across variations in milk composition, processing conditions and storage environments. This distinction is particularly important for emerging technologies, whose reported performance frequently depends on equipment configuration, treatment delivery and the endpoints selected for evaluation. Figure 3 summarises the validation domains investigated across preservation approaches, providing a framework for distinguishing laboratory observations from evidence supporting practical implementation.

**Figure 3:**
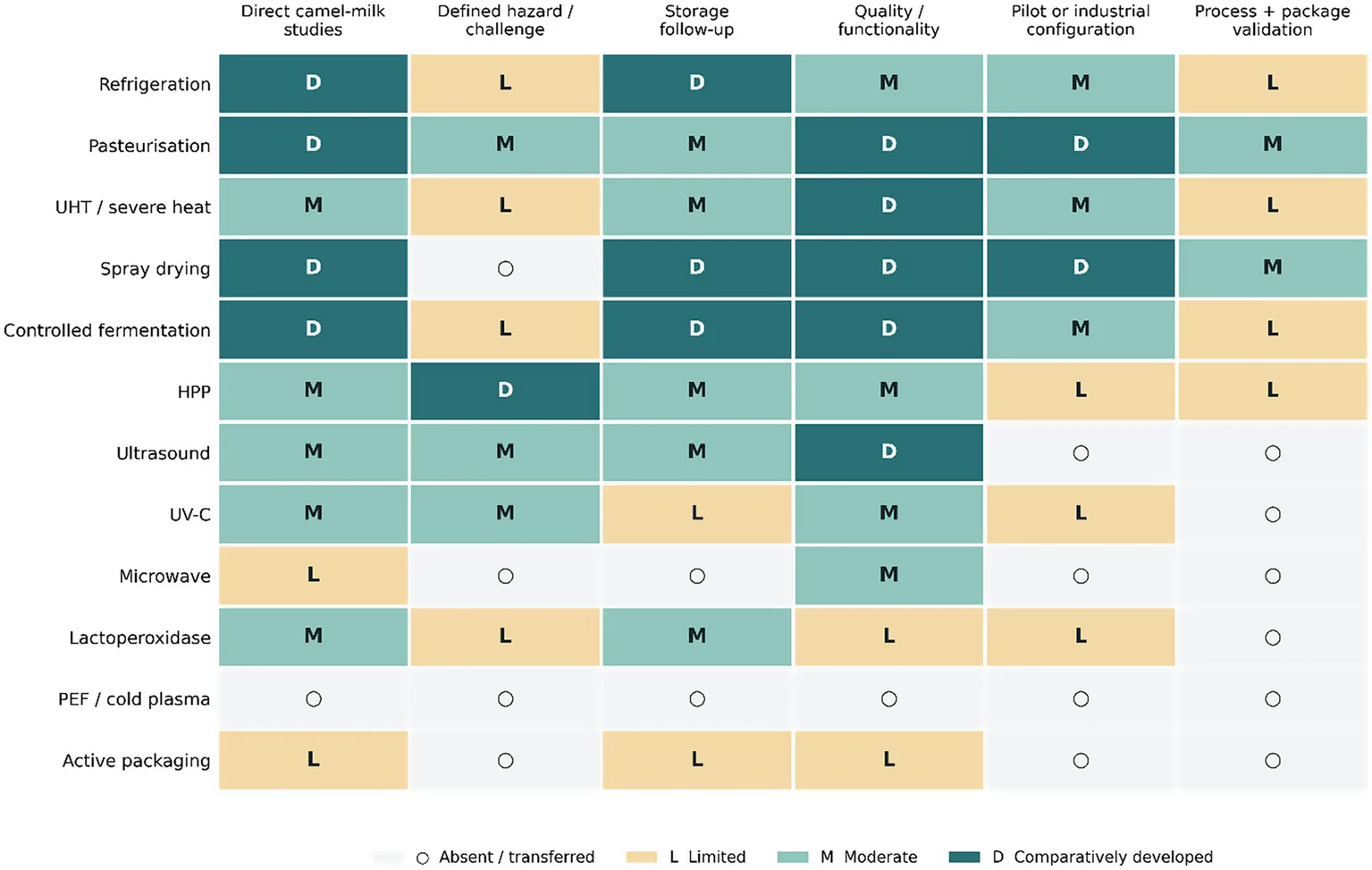
Evidence maturity across preservation technologies and validation domains based on the existence and design of camel milk studies.

### 5.1 Laboratory efficacy

Laboratory investigations provide essential information on microbial susceptibility, treatment-induced changes in milk constituents and the conditions under which preservation effects can be achieved. However, their interpretation depends on the experimental design and the relevance of the measured endpoints. A statistically significant reduction in total microbial counts demonstrates an effect under the conditions tested but does not establish control of a defined foodborne hazard. Similarly, the absence of significant changes in protein concentration, colour or other physicochemical parameters cannot independently demonstrate preservation of product quality. These distinctions become particularly important when comparing studies involving different microbial targets, treatment intensities, analytical methods and milk matrices.

The emerging-technology studies summarised in Table 2 illustrate both the value and limitations of laboratory evidence. Osaili et al. (62) investigated high-pressure processing using inoculated foodborne pathogens, multiple camel-milk batches and refrigerated storage, providing a comparatively comprehensive assessment of microbial responses and selected quality attributes. Nevertheless, the pressure–time conditions examined did not establish pasteurisation equivalence for the most resistant organism. Mudgil et al. (78) evaluated ultrasound at different nominal power settings and monitored microbial and quality attributes during refrigerated storage. Although the findings demonstrated treatment-dependent effects, reliance on background microbial counts and nominal equipment power limits their transferability to pathogen-specific validation or alternative processing configurations. Dhahir et al. (57) investigated ultrasound against defined pathogens while controlling bulk treatment temperature, but the batch configuration and prolonged exposure do not establish equivalent performance at industrial scale. These limitations do not invalidate the experimental findings; rather, they define the boundaries within which the reported effects can be interpreted.

The contrast between Tables 2, 3 illustrates why laboratory efficacy cannot be equated with validated preservation performance. The experimental studies provide different combinations of microbial, physicochemical and storage outcomes, whereas the validation framework identifies the additional evidence required to establish their reproducibility and relevance to the intended product. Several limitations recur across process classes. Studies based on indigenous microbial counts cannot establish control of a defined pathogen; experiments reporting nominal equipment settings may not adequately characterise the treatment received by the least-exposed product fraction; and immediate quality measurements do not demonstrate stability throughout the intended shelf life. Moreover, insufficient characterisation of independent replication limits conclusions regarding robustness across variable camel-milk batches. These limitations are interconnected because uncertainty in treatment delivery, matrix representativeness or microbial recovery can substantially alter the interpretation of an otherwise promising experimental result. The evidence patterns illustrated in Figure 3 should therefore be understood as differences in the validation domains investigated rather than quantitative measures of technological superiority.

A further limitation concerns the distinction between immediate treatment efficacy and subsequent product behaviour. Microorganisms surviving processing may include sublethally injured cells whose recovery depends on the enumeration method and the conditions encountered during storage. Similarly, treatment-induced modifications in protein structure, colloidal stability or oxidative status may become technologically relevant only after prolonged storage or temperature fluctuations. Immediate microbial reductions and physicochemical measurements should consequently be interpreted as components of preservation performance rather than definitive indicators of safety, stability or shelf life.

Experimental replication also determines the reliability and generalisability of laboratory findings. Repeated plating of a single treated sample estimates analytical variability, whereas independently collected and processed milk batches capture differences associated with raw-material composition and process operation. Subsamples from the same treatment run cannot be considered independent biological or process replicates. Several studies report triplicate measurements without sufficient information to establish the level of experimental independence. Such designs may provide useful estimates of analytical precision but offer limited evidence of process robustness. Accordingly, the interpretation of reported variability must distinguish analytical replication, independent milk batches and independent processing runs.

Taken together, laboratory studies establish whether a treatment produces a measurable response within a defined experimental system and identify conditions warranting further investigation. Progress towards validation requires these observations to be linked to relevant microbial targets, characterised treatment delivery, representative matrix variability and predefined quality criteria.

### 5.2 Validation criteria

Process validation requires evidence that a preservation treatment consistently achieves a specified objective under defined and reproducible operating conditions. The evidence required depends on the claim being made. Reduction of indigenous microbial counts, a defined log reduction of an inoculated pathogen, extension of refrigerated shelf life and achievement of commercial sterility represent distinct outcomes that cannot be treated as interchangeable. Pathogen-specific lethality claims require appropriately designed inactivation studies, whereas shelf-life claims additionally require storage assessments incorporating relevant microbiological, physicochemical and sensory failure criteria. Commercial sterility requires evaluation of the integrated process, hermetic packaging and product stability under the specified storage conditions. Table 3 organises these requirements according to the principal sources of uncertainty that may compromise the interpretation or transferability of experimental findings.

Validation begins with the definition of the product, the relevant hazard and the intended preservation outcome. Raw fluid milk, pasteurised milk, fermented products, concentrates and powders differ in microbial risks, physicochemical characteristics and acceptable quality limits. Target microorganisms should therefore be selected according to their relevance to the product and intended storage conditions rather than experimental convenience. Reductions in background microbial populations cannot substitute for evidence of pathogen lethality. Where direct challenge testing with the target pathogen is impractical, the use of a surrogate requires justification of its resistance characteristics, behaviour in the relevant matrix and suitability for recovery following treatment.

A second requirement is accurate characterisation of the treatment delivered to the product. Nominal equipment settings are insufficient when treatment intensity varies spatially or temporally within the processing system. Thermal validation requires the complete product time–temperature history and identification of the least-heated location. High-pressure processing requires consideration of pressure, compression and decompression profiles, treatment time and temperature. UV-C systems require characterisation of optical properties, fluence distribution and reactor hydrodynamics, while ultrasound studies must report acoustic treatment conditions, energy delivery and temperature changes. Microwave processing requires assessment of spatial temperature heterogeneity and identification of potential cold regions. These measurements are essential because surviving microorganisms may originate from product fractions receiving less severe exposure than indicated by average or nominal processing parameters.

Challenge-study design must reflect the conditions under which microbial control is expected. Inoculum preparation should account for the physiological state of the target organism without materially altering the characteristics of the milk. Strain mixtures may capture variability in treatment resistance, although individual-strain responses may be necessary to identify the most resistant component. Appropriate controls should distinguish treatment-induced inactivation from natural decline, attachment or sampling losses. Enumeration methods must account for potential recovery of sublethally injured cells, while detection limits, independent replication and confidence intervals should be reported to support the interpretation of microbial reductions. In particular, the absence of detectable survivors cannot be equated with complete inactivation without considering the analytical detection limit.

Validation must also demonstrate robustness to variation in the camel-milk matrix. Independent batches should represent the range of compositional and microbiological conditions relevant to the intended production system, including differences in total solids, fat, pH, mineral balance, optical properties and initial microbial load. Pooling milk before treatment may reduce experimental variability but prevents assessment of between-batch differences. At least three independently produced batches provide a useful starting point for investigating process-response variability, although the number required for validation depends on the expected heterogeneity, study objectives and statistical precision. Analytical replicates must be distinguished from independent production and processing runs, and statistical analyses should preserve this experimental hierarchy.

Microbial safety and product quality must be evaluated within the same validation framework. A treatment that achieves the required microbial reduction but causes unacceptable aggregation, sedimentation, oxidation or flavour deterioration cannot be considered technologically suitable for the intended product. Conversely, the absence of statistically significant quality changes does not demonstrate equivalence between treatments. Quality acceptance limits should be defined before comparison and may include sensory thresholds, sedimentation, solubility, nutrient retention or other product-specific specifications. Equivalence or non-inferiority approaches, where appropriate, provide a stronger basis for evaluating quality retention than reliance on non-significant differences alone. Assessment should also extend beyond immediate post-treatment measurements when deterioration may develop during storage.

The final validation requirements concern reproducibility under representative processing conditions, integration with packaging and verification during routine operation. Changes in equipment scale may alter residence-time distributions, heat transfer, mixing, fouling and treatment uniformity. Pilot-scale investigations should therefore evaluate the least-treated product fraction and relevant operating extremes rather than relying exclusively on average processing conditions. Critical limits, monitoring procedures, corrective actions and verification requirements must be established for the intended process. As emphasised in Table 3, these requirements are not merely reporting recommendations: they address distinct inferential errors that may arise when laboratory findings are extrapolated beyond the conditions under which they were obtained. Together, they provide the basis for progressing from experimental demonstrations to preservation claims that can be supported in commercial production.

### 5.3 Product-specific validation paths

Although the underlying principles of process validation are broadly applicable, the evidence required to substantiate preservation performance differs substantially among camel-milk products. Refrigerated fluid milk, fermented products and powders present distinct combinations of microbial hazards, physicochemical instability and storage-related failure mechanisms. Consequently, validation strategies must be defined according to the characteristics of the finished product and its intended distribution conditions rather than applying a uniform set of experimental endpoints (66, 67, 109).

For refrigerated fluid camel milk, validation must connect the initial microbiological quality of the raw material with the efficacy of the preservation treatment, hygienic filling, packaging performance and subsequent temperature control. The initial microbial load and the time elapsed before refrigeration influence both the population exposed to treatment and the potential accumulation of heat-stable spoilage enzymes. Accordingly, a defined microbial reduction does not necessarily translate into an equivalent extension of shelf life across milk batches with different handling histories. Validation should therefore address relevant pathogens, spoilage microorganisms and quality deterioration throughout the intended storage period, including foreseeable temperature deviations.

For fermented camel-milk products, preservation performance depends on interactions among starter-culture activity, acidification kinetics, microbial inhibition and the development of acceptable product structure. Validation should establish whether the selected culture consistently achieves the intended acidification profile and controls relevant hazards under representative processing and storage conditions. Post-acidification, survival of undesirable microorganisms, yeast and mould growth, syneresis and texture deterioration must also be considered. Because milk composition, starter selection and formulation can alter both microbial behaviour and gel formation, evidence obtained from one fermented product cannot be assumed to apply to another without further assessment.

Camel-milk powders require a different validation approach because reducing water activity restricts microbial growth without necessarily eliminating viable microorganisms. The relevant evidence must connect the microbial effects of processing with powder formation, residual moisture, water activity, packaging barriers and stability during storage. Moisture uptake, lactose crystallisation, caking, oxidation and deterioration of reconstitution performance may determine product failure even when immediate microbiological and compositional specifications are satisfied. Validation should therefore consider the complete processing and storage system, including oxygen and moisture transmission through packaging and physicochemical changes occurring under the intended distribution conditions (66, 67, 109).

Selection of representative worst-case conditions is equally product dependent. Elevated solids or fat contents may influence microbial resistance during some treatments, whereas reduced optical transmission is particularly relevant to UV-C processing. High initial microbial loads can shorten refrigerated shelf life despite comparable treatment-induced reductions. Variations in pH and mineral equilibrium may increase the risk of protein aggregation during thermal or pressure processing. Independent milk batches are therefore informative only when they encompass the sources of variation most likely to affect the specific preservation outcome being validated (109, 110).

The transition from laboratory to pilot or industrial processing introduces additional sources of heterogeneity. Continuous thermal systems exhibit distributions of residence time and temperature; UV-C reactors generate non-uniform fluence exposure; ultrasound produces spatially heterogeneous acoustic fields; and microwave systems may develop uneven electromagnetic energy deposition and temperature profiles. These differences affect the least-treated product fraction, which may determine residual microbial survival. Consequently, pilot-scale validation should characterise limiting exposure conditions, including the effects of fouling, equipment configuration and end-of-run operation, rather than assuming that equivalent average settings produce equivalent microbial outcomes (66, 67, 110).

The product distinctions documented in Supplementary Table S1 reinforce the need to interpret the validation requirements in Table 3 within the specific matrix investigated. Evidence obtained from raw or pasteurised fluid milk cannot be transferred directly to fermented products, cheese systems, whey or powders because processing modifies the physicochemical environment governing microbial behaviour and quality-related reactions. For refrigerated milk, the critical evidence concerns microbial control and deterioration during storage; for fermented products, it additionally involves acidification kinetics, microbial interactions and structural stability; and for powders, it extends to water activity, moisture sensitivity and reconstitution performance. The evidence inventory also identifies differences in camel species and instances in which species was not reported, introducing further uncertainty when transferring processing conditions between studies. These distinctions demonstrate that validation must be organised around the intended product and preservation claim rather than extrapolated from the apparent effectiveness of a technology in another camel-milk matrix.

Overall, the available evidence indicates that emerging camel-milk preservation technologies have not yet been supported by complete, publicly documented industrial validation packages. This limitation should not be interpreted as evidence of technological ineffectiveness. Rather, it highlights the need for investigations integrating defined microbial targets, measured treatment delivery, representative milk variability, quality retention, storage performance and pilot-scale reproducibility. Comparative studies conducted at equivalent microbial lethality, using common milk batches and product-relevant quality endpoints, would provide a stronger basis for determining which preservation strategies can be reliably transferred to commercial camel-milk processing.

## 6 Future prospects for industrial research and implementation

The future development of camel-milk preservation technologies will depend less on identifying additional processing alternatives than on establishing conditions under which existing and emerging approaches can deliver reproducible microbial control while maintaining product quality and industrial feasibility. The most promising research directions are therefore those that connect matrix-specific processing responses with the operational constraints of camel-milk production and distribution. This requires moving beyond demonstrations of treatment efficacy towards integrated process designs that account for raw-milk variability, product characteristics, equipment performance, storage conditions and the infrastructure available to processors.

Industrial opportunities differ substantially across production systems. In decentralised pastoral chains, improvements in hygienic milking, rapid cooling, shorter transport times and controlled fermentation are likely to provide more immediate benefits than the introduction of equipment requiring sophisticated process monitoring. Where refrigeration is unavailable or delayed, temporary antimicrobial control may have a complementary role, provided its application is validated and permitted under the relevant regulatory framework. Small-scale processors may benefit most from reliable batch pasteurisation, hygienic filling and refrigerated distribution, whereas centralised facilities are better positioned to investigate continuous thermal processing, UHT treatment, drying, high-pressure processing (HPP) and engineered UV-C systems. These distinctions suggest that industrial research should prioritise technologies according to their suitability for specific production chains rather than their novelty or reported laboratory performance.

A major research opportunity lies in establishing comparable operating windows for conventional and emerging technologies. Studies conducted with common camel-milk batches, defined microbial targets and equivalent lethality objectives would allow more meaningful comparisons among thermal processing, HPP, UV-C and thermosonication. Such investigations should assess microbial inactivation alongside protein stability, oxidation, sensory quality and subsequent storage behaviour, thereby identifying processing conditions that balance safety and product acceptability. Equally important is the development of composition–process relationships capable of predicting how variations in pH, calcium activity, protein concentration, fat content and optical attenuation influence treatment performance. Integrating these relationships into process models could support the identification of operating limits that remain effective across the compositional variability encountered in commercial camel-milk production.

Another priority is to establish how processing-induced changes influence product stability beyond the immediate treatment period. The recovery of sublethally injured microorganisms, persistence of extracellular enzymes and progressive development of physicochemical defects may determine shelf-life failure even when initial microbial and quality measurements are satisfactory. Longitudinal studies that combine microbial recovery, enzymatic activity, colloidal stability, oxidation and sensory deterioration under representative storage conditions would help establish the relationship between treatment intensity and actual product performance. This approach is particularly relevant to refrigerated fluid milk, fermented products and powders, for which the mechanisms and consequences of deterioration differ substantially.

The transition to industrial application also requires research at pilot scale under representative operating conditions. Process performance should be evaluated in relation to throughput, energy requirements, treatment uniformity, fouling, cleaning procedures, packaging compatibility and operational reliability. For emerging technologies, these investigations are essential to determine whether the effects observed in laboratory systems can be maintained as equipment geometry, processing volume and operating duration change. Pulsed electric fields, cold plasma, active packaging and antimicrobial nanoformulations remain legitimate research directions, but their industrial potential cannot yet be established from the available camel-milk evidence. Their investigation should therefore be guided by clearly defined product applications and measurable advantages over established preservation approaches, rather than by technological novelty alone.

Economic and environmental performance represents a further gap in the evidence. None of the direct studies included in this review provides a comprehensive comparison of preservation technologies at equivalent levels of microbial safety and product quality. Although drying can reduce the mass transported by removing water, it requires energy-intensive concentration and dehydration operations. UHT processing may reduce dependence on refrigerated distribution but introduces more severe thermal conditions and specific packaging requirements, while HPP generally involves substantial capital investment and continued refrigerated storage. These considerations identify potential trade-offs rather than demonstrated advantages. Comparative techno-economic and environmental assessments incorporating capital expenditure, energy consumption, throughput, cleaning, product losses, packaging and distribution requirements are needed before industrial recommendations can be made on the basis of cost or sustainability.

Regulatory and institutional developments will be equally important in translating research outcomes into commercial practice. Camel-milk-specific evidence is needed to support product standards, microbiological criteria, heat-treatment verification and substantiated shelf-life claims. In particular, processing indicators and operating limits developed for bovine milk should not be adopted without demonstrating their suitability for camel-milk matrices. Collaboration among researchers, producers, processors, equipment manufacturers and regulatory authorities could facilitate the development of validation protocols, process specifications and monitoring procedures adapted to different production systems.

Ultimately, the industrial promise of camel-milk preservation research lies in developing processes that are not only microbiologically effective but also robust to matrix variability, compatible with product-quality requirements and feasible within the infrastructure of the intended production chain. The most valuable advances are likely to emerge from integrated studies combining microbial challenge testing, measured treatment delivery, quality and shelf-life assessment, pilot-scale operation and economic evaluation. Such evidence would provide a stronger foundation for selecting and implementing preservation strategies that support the safe and sustainable expansion of the camel dairy sector.

## 7 Conclusion

The evidence synthesised in this review identifies the interaction between camel-milk matrix characteristics, processing conditions, microbial efficacy and product-quality requirements as a central determinant of preservation performance. Compositional variability, protein organisation, mineral equilibrium and initial microbial quality influence treatment responses and subsequent product stability, limiting the direct transfer of processing conditions established for bovine milk or other camel-milk products. Refrigeration, controlled fermentation, pasteurisation and drying remain established preservation approaches, while high-pressure processing, UV-C, ultrasound and rapid thermal technologies present additional opportunities for specific applications. However, differences in experimental designs, microbial targets, treatment delivery and analytical endpoints preclude a reliable overall ranking of these technologies.

The critical synthesis highlights a persistent gap between demonstrated laboratory efficacy and evidence supporting validated preservation processes. Although selected studies provide pathogen-specific inactivation data, important limitations remain in the characterisation of delivered treatment, assessment of sublethal microbial injury, representation of independent milk batches, evaluation of storage stability and demonstration of process reproducibility under industrially relevant conditions. The heterogeneity of the available studies, together with incomplete reporting of camel species, product characteristics and processing parameters, further restricts cross-study comparisons and the transferability of reported outcomes. Accordingly, the evidence classification and validation framework presented in this review characterise the scope and maturity of published findings without implying pooled treatment effects or formal risk-of-bias assessments.

Advancing camel-milk preservation requires product-specific validation strategies that integrate defined microbial targets, representative matrix variability, measured treatment delivery and quality assessment throughout the intended shelf life. Combining these elements with appropriate processing and distribution controls can support the development of reproducible preservation systems adapted to the technological characteristics of camel milk and the operational conditions of its production chains.
