# Supplementary material for: Camel milk preservation: matrix effects across conventional and emerging technologies
Source: Front Nutr. 2026 Oct 23;13:1990078. doi: 10.3389/fnut.2026.1990078 (PMC13740251; doi:10.3389/fnut.2026.1990078)
Supplement: Supplementary file 1 [file Table_1.docx]

Table S1. Evidence-source inventory supporting matrix, process, product and validation interpretations. Evidence category is assigned as direct, supportive or transferred according to the operational definitions in Section 2.

| **Reference** | **Camel species** | **Milk/product matrix** | **Process/design and scale** | **Principal endpoint** | **Evidence category** | **Role in this review** |
| --- | --- | --- | --- | --- | --- | --- |
| Farah and Atkins (1992) | Camelus dromedarius | Milk | Severe laboratory heating | Heat coagulation and pH dependence | Direct | matrix mechanism |
| Abdurahman (1995) | Camelus dromedarius | Raw milk | Udder-health markers | NAGase and serum albumin | Supportive | raw-material evidence |
| Abu-Tarboush (1996) | Camel species not reported; bovine comparator | Whole milk | Yogurt starter fermentation | Growth and proteolysis | Direct | fermentation evidence |
| Elagamy (2000) | Camelus dromedarius | Milk | Graded heat treatment | Antimicrobial-protein activity | Direct | quality/mechanism evidence |
| Attia et al. (2001) | Camelus dromedarius | Milk | Lactic fermentation | Microbiology and rheology | Direct | product evidence |
| Lore et al. (2005) | Camel species not reported | Suusac | Traditional fermentation | Culture-dependent microbiota | Supportive | fermentation evidence |
| El-Hatmi et al. (2006) | Camelus dromedarius | Colostrum and milk | Protein characterisation | IgG, alpha-lactalbumin, albumin, lactoferrin | Supportive | matrix evidence |
| Konuspayeva et al. (2007) | C. dromedarius, C. bactrianus and hybrids | Milk | Protein characterisation | Lactoferrin and immunoglobulins | Supportive | matrix/species evidence |
| Seifu (2007) | Camelus dromedarius | Milk and products | Field handling and preservation | Practices and utilisation | Supportive | implementation evidence |
| Wernery et al. (2008) | Camel species not reported | Milk | Graded thermal treatment | ALP, GGT and LPO | Direct | verification evidence |
| Hashim et al. (2009) | Camelus dromedarius | Yogurt | Set-type fermentation | Quality and acceptability | Direct | product evidence |
| Kamau et al. (2010) | Camel species not reported | Raw milk | Lactoperoxidase activation during storage | Keeping quality | Direct | temporary-hurdle evidence |
| Lorenzen et al. (2011) | Camel species not reported | Raw and pasteurised milk | Thermal treatment | Indigenous enzyme activities | Direct | verification evidence |
| Shori (2012) | Camel species varies or not reported | Traditional fermented milks | Traditional fermentation | Composition and microbiota | Supportive | fermentation evidence |
| Konuspayeva et al. (2014) | Camelus dromedarius | Milk | Cheese-processing adjustments | Coagulation behaviour | Direct | functionality evidence |
| Lü et al. (2014) | Camel species not reported | Shubat isolate | Bacteriocin purification | Antimicrobial activity | Supportive | antimicrobial mechanism |
| Al-Zoreky and Al-Otaibi (2015) | Camel species not reported | Milk | Yogurt manufacture | Fermentation and product quality | Direct | product evidence |
| Felfoul et al. (2015) | Camel species not reported; bovine comparator | Whey | Laboratory heating | Protein deposition | Direct | matrix mechanism |
| Ibrahim (2015) | Camel species not reported | Fermented milk | EPS-producing starters | Rheology and sensory properties | Direct | formulation evidence |
| El Hatmi et al. (2016) | Camel species not reported | Fermented milk | Peptide identification | Bioactive peptides | Direct | mechanistic evidence |
| Fguiri et al. (2016) | Camel species not reported | Raw-milk isolates | Starter-culture screening | LAB identity and technological traits | Supportive | starter evidence |
| Ibrahem and El Zubeir (2016) | Camel species not reported; ovine comparator | Milk and mixed milk | Yogurt processing | Composition and sensory quality | Direct | product evidence |
| Abusheliabi et al. (2017a) | Camel species not reported | Milk | Pathogen-growth study | Foodborne-pathogen inhibition | Direct | microbial evidence |
| Abusheliabi et al. (2017b) | Camel species not reported | Raw and pasteurised milk | Defined-temperature storage | Cronobacter kinetics | Direct | microbial evidence |
| Amenu et al. (2017) | Camel species not reported; bovine comparator | Milk | Lactoperoxidase activation | Acidification, counts and shelf life | Direct | hurdle evidence |
| Benabdelkamel et al. (2017) | Camelus dromedarius | Whey | Multiple heat treatments | Proteomic response | Direct | quality evidence |
| Felfoul et al. (2017) | Camel species not reported; bovine comparator | Milk | Heat treatment | Proteomic response | Direct | mechanism evidence |
| Kamal et al. (2017) | Camel species not reported; bovine comparator | Milk | Heating plus calcium/phosphate | Rennet coagulation | Direct | functionality evidence |
| Ahmad et al. (2018) | Camel species not reported | Whey powder | Gamma irradiation | Antibody and protein stability | Direct | irradiation-quality evidence |
| Mudgil et al. (2018) | Camel species not reported | Yogurt | Gelatin formulation | Rheology, microstructure and sensory quality | Direct | product evidence |
| Ryskaliyeva et al. (2018) | C. dromedarius, C. bactrianus and hybrids | Milk | Proteomic characterisation | Protein-fraction variability | Supportive | matrix/species evidence |
| Alaoui Ismaili et al. (2019) | Camelus dromedarius | Raw milk | Field sampling | Composition and microbial quality | Supportive | raw-material evidence |
| Genene et al. (2019) | Camelus dromedarius | Milk | Heat treatments | Whey denaturation and rennetability | Direct | quality evidence |
| Ho et al. (2019) | Camel species not reported | Spray-dried camel milk powder | Accelerated storage | Physicochemical powder stability | Direct | storage evidence |
| Amrouche et al. (2020) | Camelus dromedarius | Raw milk | Microbiota characterisation | Community composition | Supportive | raw-material evidence |
| Dhahir et al. (2020) | Camel species not reported | Pasteurised inoculated milk | Probe ultrasound; 70 mL | Pathogens, proteins, lipids and volatiles | Direct | challenge evidence |
| El-Zubeir (2020) | Camel species not reported | Milk | Lactoperoxidase activation and storage | Microbial loads | Direct | hurdle evidence |
| Lund et al. (2020) | Camel species not reported | Milk | Heating and storage | Shelf life and sensory response | Direct | thermal/storage evidence |
| Mbye et al. (2020) | Camel species not reported; bovine comparator | Cheese milk | Cheese manufacture | Coagulation, composition and sensory quality | Direct | product evidence |
| Mohamed et al. (2020) | Camelus dromedarius | Milk | Capillary electrophoresis | Casein and alpha-lactalbumin | Supportive | matrix evidence |
| Rahmeh et al. (2020) | Camel species not reported | Raw-milk isolate and model food system | Enterococcus antimicrobial | Pathogen inhibition | Supportive | antimicrobial evidence |
| Algboory and Muhialdin (2021) | Camel species not reported | Fermented milk | L. plantarum IS10 | Antimicrobial peptides and challenge response | Direct | mechanistic evidence |
| Aralbayev et al. (2021) | Camel species not reported | Whole milk | Spray drying optimisation | Process and powder parameters | Direct | process evidence |
| Dhahir et al. (2021) | Camel species not reported | Pasteurised inoculated milk | Continuous-flow UV-C | Pathogens and quality | Direct | challenge evidence |
| Kadri et al. (2021) | Camel species not reported | Raw milk | Microbiota characterisation | Bacterial diversity | Supportive | raw-material evidence |
| Mbye et al. (2021) | Camel species not reported; bovine comparator | Cheese | Pasteurisation and HPP | Cheese quality and proteolysis | Direct | process/product evidence |
| Mohamed Abdalla (2021) | Camel species not reported | Pasteurised milk | Packaging comparison during storage | Shelf life | Direct | package evidence |
| Perusko et al. (2021) | Camel species not reported | Milk powder | Spray drying | Maillard products | Direct | quality evidence |
| Seligsohn et al. (2021) | Camelus dromedarius | Milk | Udder-health assessment | SCC, NAGase and LDH | Supportive | raw-material evidence |
| Sobti et al. (2021) | Camel species not reported; bovine comparator | Laban | Pectin/alginate formulation | Gel and product properties | Direct | formulation evidence |
| Zahra et al. (2021) | Camelus dromedarius, Targui breed | Raw milk | Field sampling | Physicochemical and microbiological quality | Supportive | raw-material evidence |
| Zhadyra et al. (2021) | Camel species not reported | Fermented milk | Culture-dependent/independent profiling | Bacterial diversity | Supportive | fermentation evidence |
| Bao et al. (2022) | Camelus bactrianus | Raw and fermented milk | Microbiome profiling | Bacterial/fungal diversity | Supportive | fermentation evidence |
| Chethouna et al. (2022) | Camel species not reported | Raw and pasteurised milk | Pasteurisation comparison | Counts and physicochemistry | Direct | process evidence |
| Mbye et al. (2022) | Camel species not reported | Cheese systems | Coagulants and processing | Texture and functionality | Direct | product evidence |
| Mudgil et al. (2022) | Camel species not reported | Milk; 100 mL | Ultrasound at several nominal powers | Background flora, proteins and storage | Direct | process evidence |
| Wang et al. (2023) | Camel species not reported | Milk | Multiple heat treatments | Maillard products and volatiles | Direct | quality evidence |
| Zhang et al. (2023) | Camel species not reported; bovine comparator | Milk | Microwave heating | Structure and composition | Direct | physicochemical evidence |
| Ahmed et al. (2024) | Camel species not reported | Raw milk | 16S rRNA PCR and sequencing | Lactic acid bacteria and contaminating taxa | Supportive | camel-specific microbial ecology |
| Wang et al. (2024) | Camel species not reported | Raw milk | Defined storage conditions | Quality deterioration | Direct | storage evidence |
| Yao et al. (2024) | Multiple mammalian species, including camel | Milk | Heat treatment | Oligosaccharide profile | Supportive | comparative evidence |
| Arain et al. (2025) | Camel species not reported | Milk | 63 degrees C, 30 min; plate-and-frame | Composition and nutrients | Direct | industrial-quality evidence |
| Chelladhurai et al. (2025) | Camel species not reported; bovine comparator | Low-fat milk | Three cultures; 28-42 degrees C; 96 h | Growth, acidification and proteolysis | Direct | fermentation kinetics |
| Farid et al. (2025) | Camelus dromedarius | Raw milk | 4 degrees C; 29 d | Counts and composition | Direct | storage evidence |
| Osaili et al. (2025) | Camel species not reported | Milk | HPP; 300-350 MPa | Pathogens, flora, storage and proteins | Direct | challenge evidence |
| Boukria et al. (2026) | Camel species not reported; caprine and bovine comparators | Fermented milk | Ultrasound before, after or before and after fermentation | Physicochemical and mineral-related responses | Direct | matrix-dependent fermentation evidence |
| Chehmat et al. (2026) | Non-camel dairy evidence | Milk systems | Critical synthesis of ozone transfer and reactivity | Dose transfer, microbial response and quality | Transferred | matrix-reactivity interpretation |
| El Kindoussy et al. (2026b) | Camelus dromedarius | Raw Moroccan camel milk | Controlled ultrasound; 5–15 min | Microbial load, colloidal structure and functionality | Direct | process and matrix-response evidence |
| Kang et al. (2026) | Camel species not reported | Milk powder | Spray and low-temperature vacuum drying | Microstructure and proteins | Direct | drying-quality evidence |

*Note:* ALP, alkaline phosphatase; EPS, exopolysaccharide; GGT, gamma-glutamyl transferase; HPP, high-pressure processing; IgG, immunoglobulin G; LAB, lactic acid bacteria; LDH, lactate dehydrogenase; LPO, lactoperoxidase; NAGase, N-acetyl-beta-D-glucosaminidase; SCC, somatic cell count; UV-C, ultraviolet-C. “Supportive” records inform matrix, microbial ecology or mechanism but do not independently validate a preservation process.
